# T Cell-Intrinsic CDK6 Is Dispensable for Anti-Viral and Anti-Tumor Responses *In Vivo*

**DOI:** 10.3389/fimmu.2021.650977

**Published:** 2021-06-24

**Authors:** Klara Klein, Agnieszka Witalisz-Siepracka, Dagmar Gotthardt, Benedikt Agerer, Felix Locker, Reinhard Grausenburger, Vanessa Maria Knab, Andreas Bergthaler, Veronika Sexl

**Affiliations:** ^1^ Institute of Pharmacology and Toxicology, University of Veterinary Medicine Vienna, Vienna, Austria; ^2^ Department of Pharmacology, Physiology and Microbiology, Division Pharmacology, Karl Landsteiner University of Health Sciences, Krems, Austria; ^3^ CeMM Research Center for Molecular Medicine of the Austrian Academy of Sciences, Vienna, Austria; ^4^ Institute of Physiology, Pathophysiology and Biophysics, University of Veterinary Medicine, Vienna, Austria

**Keywords:** CDK6, CD8+ T cells, metabolism, interferon signaling, suppressor of cytokine signaling (SOCS), anti-viral response, anti-tumor response

## Abstract

The cyclin-dependent kinase 6 (CDK6) regulates the transition through the G1-phase of the cell cycle, but also acts as a transcriptional regulator. As such CDK6 regulates cell survival or cytokine secretion together with STATs, AP-1 or NF-κB. In the hematopoietic system, CDK6 regulates T cell development and promotes leukemia and lymphoma. CDK4/6 kinase inhibitors are FDA approved for treatment of breast cancer patients and have been reported to enhance T cell-mediated anti-tumor immunity. The involvement of CDK6 in T cell functions remains enigmatic. We here investigated the role of CDK6 in CD8+ T cells, using previously generated CDK6 knockout (*Cdk6* ^-/-^) and kinase-dead mutant CDK6 (*Cdk6* ^K43M^) knock-in mice. RNA-seq analysis indicated a role of CDK6 in T cell metabolism and interferon (IFN) signaling. To investigate whether these CDK6 functions are T cell-intrinsic, we generated a T cell-specific CDK6 knockout mouse model (*Cdk6* ^fl/fl^ CD4-Cre). T cell-intrinsic loss of CDK6 enhanced mitochondrial respiration in CD8+ T cells, but did not impact on cytotoxicity and production of the effector cytokines IFN-γ and TNF-α by CD8+ T cells *in vitro*. Loss of CDK6 in peripheral T cells did not affect tumor surveillance of MC38 tumors *in vivo*. Similarly, while we observed an impaired induction of early responses to type I IFN in CDK6-deficient CD8+ T cells, we failed to observe any differences in the response to LCMV infection upon T cell-intrinsic loss of CDK6 *in vivo*. This apparent contradiction might at least partially be explained by the reduced expression of *Socs1*, a negative regulator of IFN signaling, in CDK6-deficient CD8+ T cells. Therefore, our data are in line with a dual role of CDK6 in IFN signaling; while CDK6 promotes early IFN responses, it is also involved in the induction of a negative feedback loop. These data assign CDK6 a role in the fine-tuning of cytokine responses.

## Introduction

Cyclin-dependent kinase 6 (CDK6) and its homolog CDK4 are serine/threonine kinases that regulate cell cycle progression. Upon binding to D-type cyclins, they promote cell cycle transition from G1- to S-phase in response to external signals, including growth factors and mitogens (1–4). Despite being redundant for cell cycle regulation, CDK4 and CDK6 have unique roles. In mice, only CDK6 deficiency is associated with defects in the hematopoietic system, including mild anemia and reduced thymocyte numbers (3–7). CDK6 kinase activity plays an important role in Notch-dependent thymocyte development and was shown to be required for Akt-driven thymocyte transformation (5–7). Moreover, CDK6 is amplified in a wide range of hematopoietic tumors, where it exerts proto-oncogenic functions (3, 4, 6, 8–14). In cooperation with transcription factors, including STAT3, AP-1 and NF-κB, CDK6 acts as a transcriptional regulator, only partially dependent on its kinase activity. CDK6-regulated genes include tumor-suppressors, tumor-promoters and inflammatory cytokines, adding complexity to the role of CDK6 during homeostasis and tumorigenesis (8–10, 12, 13, 15).

CDK4/6 kinase inhibitors have been effective in clinical trials and gained FDA approval for the treatment of hormone-receptor positive breast cancer (16–23). CDK4/6 inhibitors not only induce growth arrest of tumor cells, but also enhance T cell-mediated anti-tumor immunity (24–27). These effects were attributed to enhanced antigen processing and presentation by tumor cells and antigen-presenting cells (APCs) in the tumor microenvironment. This increases tumor immunogenicity and thus CD8+ T cell-mediated anti-tumor immunity (24, 25, 27). In addition, CDK4/6 inhibitors were reported to directly affect T cells and to increase cytokine production by enhancing NFAT signaling (25, 27, 28). Increased IL-2 production in the CD4+ Jurkat T cell line was recapitulated by knockdown of CDK6, but not CDK4, in line with the privileged role of CDK6 in T cells (25). Furthermore, regulatory T cells (Tregs) are highly sensitive to CDK4/6 inhibitor-mediated anti-proliferative effects, which contributes to the enhanced tumor surveillance (24). These data indicate an important role of the immune system for the anti-cancer effects of CDK4/6 inhibitors (24–27).

As CDK6 plays an important role in T cell development and CDK4/6 inhibitors boost T cell-mediated anti-tumor immunity, we here aimed to delineate T cell-intrinsic functions of CDK6 focusing on peripheral CD8+ T cells. Using a novel T cell-specific conditional CDK6 knockout mouse model, we found that CD8+ T cells depend on CDK6 for expansion *in vitro* and show higher mitochondrial respiration upon loss of CDK6. This is not associated with an altered effector function of CDK6-deficient CD8+ T cells *in vitro*, apart from a slight increase in IL-2 production. In addition, T cell-intrinsic loss of CDK6 does not affect anti-viral and anti-tumor responses *in vivo.* Our data provide first insights into a potential dual function of CDK6 in regulating type I IFN signaling in CD8+ T cells, promoting both interferon-stimulated gene (ISG) expression but also negative feedback loops.

## Materials And Methods

### Mice and Cell Lines

Wild-type (C57BL/6), *Cdk6* ^-/-^ (C57BL/6 background) (5) and *Cdk6* ^K43M^ (C57BL/6 background) (7) were bred in house. The T cell-specific mouse model was obtained by crossing *Cdk6* ^fl/fl^ [C57BL/6N-Cdk6^tm1c(EUCOMM)Wtsi^/Tcp (29)] to CD4-Cre mice [B6;D2-Tg(Cd4-cre)1Cwi/CwiCnrm (30), bred on C57BL/6 background and kindly provided by Wilfried Ellmeier, Institute of Immunology, MedUni Vienna]. All mice were maintained under specific pathogen-free conditions at the University of Veterinary Medicine Vienna according to Federation for Laboratory Animal Science Associations (FELASA) guidelines (2014). In all experiments, sex and age matched (8-10 weeks old) mice were used. Animal experiments were approved by the Ethics and Animal Welfare Committee of the University of Veterinary Medicine Vienna and the national authority (Austrian Federal Ministry of Science and Research) according to §§ 26ff. of Animal Experiments Act, Tierversuchsgesetz 2012—TVG 2012, under licenses BMBWF-68.205/0174-V/3b/2018 (GZ 2020-0.050.677) and BMWFW-66.009/0361-WF/V/3b/2017 (GZ 2021-0.009.992), and were conducted according to the guidelines of FELASA and ARRIVE.

The FcγR+ murine mastocytoma cell line P815 (31, 32) (kindly provided by Wilfried Ellmeier, Institute of Immunology, MedUni Vienna) and the murine colon carcinoma cell line MC38 (33, 34) were cultured in DMEM (Sigma) complete medium containing 10% FCS (Bio & Sell), 100 U/mL penicillin (Sigma), 100 mg/mL streptomycin (Sigma), and 50 μM 2-mercaptoethanol (Sigma).

### RNA-Sequencing

Splenic CD3+CD8+ T cells were *ex vivo* sorted from 8-10 week-old male WT, *Cdk6^-^* ^/-^ and *Cdk6* ^K43M^ mice on a BD FACSAria Sorter. Two mice were pooled for each of the three biological replicates per genotype. 10^6^ sorted T cells were lysed in RLT buffer and RNA was isolated using the RNeasy Mini kit (Qiagen). RNA-sequencing was performed at the Research Center for Molecular Medicine (CeMM). RNA-seq libraries were prepared with the TruSeq Stranded mRNA LT sample preparation kit (Illumina). Libraries were quantified with the Qubit 2.0 Fluorometric Quantitation system (Thermo Fisher Scientific) and assessed by the Experion Automated Electrophoresis System (BioRad). Single-end, 50 bp sequencing of pooled libraries was performed on HiSeq 3000/4000 instruments (Illumina, San Diego, CA, USA). Base calls, obtained by the real-time analysis software (Illumina), were converted into the multiplexed, unaligned BAM format before demultiplexing into sample-specific, unaligned BAM files. After quality control of raw data with FastQC and removement of adapters and low-quality reads with Trimmomatic (version 0.36), reads were mapped to the GENECODE M13 genome using STAR (version 2.5.2b) with default parameters. Counts for union gene models were obtained using featureCounts from the Subread package (version 1.5.1). Differentially expressed (FDR < 0.05 and fold change < -1.25 or > 1.25) genes were identified using edgeR (version 3.30.3). The RNA-seq data reported in this paper have been deposited in the Gene Expression Omnibus database (Accession ID: GSE164002). Data is not yet available but will be made available upon request.

Pathway overrepresentation analysis (pantherdb.org) was performed using Reactome Pathways as annotation data set. Out of the significant pathways (FDR <0.05), only non-redundant terms were considered. In the case of redundant pathways including the same set of enriched genes, the more significant pathway (lower FDR) was considered. Type I and II interferon-stimulated genes (ISGs) were extracted from the interferome.org database. From those genes, the “core type I ISGs” (130 genes) were defined by combining the top 100 upregulated genes upon IFN-α with the top 100 upregulated genes upon IFN-β treatment from murine datasets. The “core type II ISGs” were defined as the top 100 upregulated genes upon IFN-γ treatment from murine datasets.

### ChIP-Sequencing Analysis

The CDK6 ChIP-seq experiment in p185^BCR-ABL^-transformed cell lines, expressing HA-tagged CDK6, has previously been performed using an antibody against HA and deposited in the Gene Expression Omnibus (GEO) database (Accession ID: GSE113752) (12). ChIP-seq peaks from HA-CDK6 expressing wild-type (WT) cell lines WT1-3, corresponding inputs and consensus peak regions are shown in **Figure 7**. Consensus peak regions were defined as ChIP-seq peak regions present in at least 2 replicates and the final consensus peak regions were obtained by subtracting the consensus peak regions of a negative control anti-HA ChIP-seq in CDK6-defcient cell lines from the WT consensus peaks to exclude unspecifically enriched regions.

### T Cell Isolation, Culture and Stimulation

Naive CD8+ T cells were isolated from splenocytes using the MagniSort Mouse CD8 Naïve T cell Enrichment Kit (Thermo Fisher Scientific). 4*10^5^ isolated CD8+ T cells were seeded per well of a 48 well plate (Sarstedt) coated with 1.5 µg/ml αCD3 (clone 145-2C11; BD Biosciences) and 2 µg/ml αCD28 (clone 37.51; BD Biosciences) and cultured for 48h in presence of 10ng/ml murine IL-2 [kindly provided by Peter Steinlein, Research Institute of Molecular Pathology (IMP)] in RPMI complete [(10% FCS (Bio & Sell), 100 U/mL penicillin (Sigma), 100 mg/mL streptomycin (Sigma), 50 μM 2-mercaptoethanol (Sigma), 1x non-essential amino acids (NEAA) (Sigma), 1mM Sodium pyruvate (Sigma)]. After 48h T cells were transferred to uncoated wells and cultured in presence of 50 ng/ml murine IL-2. T cell numbers were analyzed by flow cytometry. For analysis of activation-induced cell death, CD8+ T cells were cultured for four days and restimulated on wells coated with 2 µg/ml αCD3/αCD28 or left unstimulated for 24h in presence of IL-2. For analysis of cytokine production by flow cytometry, CD8+ T cells cultured for six days were stimulated with Cell Activation Cocktail, containing phorbol-12-myristate 13-acetate (PMA), ionomycin and Brefeldin A (Biolegend) or only Brefeldin A as a control for 4h.

For the analysis of proliferation dye dilution, isolated naïve CD8+ T cells were stained with 1µM carboxyfluorescein diacetate succinimidyl ester (CFSE, Molecular Probes, CellTrace CFSE Cell Proliferation Kit) according to manufacturer’s instructions. Labelled CD8+ T cells were cultured as described above and CFSE dye dilution was analyzed by flow cytometry after 72h.

Naïve CD4+ T cells were sorted from peripheral organs [spleens and peripheral lymph nodes (pLNs)] on a BD FACSAria Sorter. 3*10^5^ CD4+ T cells were seeded per well of a 48 well plate (Sarstedt) coated with 1 µg/ml αCD3 (clone 145-2C11; BD Biosciences) and 3 µg/ml αCD28 (clone 37.51; BD Biosciences) and cultured for 72h in presence of 10ng/ml murine IL-2 [kindly provided by Peter Steinlein, Research Institute of Molecular Pathology (IMP)] in RPMI complete [10% FCS (Bio & Sell), 100 U/mL penicillin (Sigma), 100 mg/mL streptomycin (Sigma), 50 μM 2-mercaptoethanol (Sigma), 1x non-essential amino acids (NEAA) (Sigma), 1 mM Sodium pyruvate (Sigma)]. For the analysis of cytokine production by flow cytometry, CD4+ T cells cultured for 72h were stimulated with Cell Activation Cocktail, containing phorbol-12-myristate 13-acetate (PMA), ionomycin and Brefeldin A (Biolegend) or only Brefeldin A as a control for 4h.

### Flow Cytometry

For blood analysis, heparin-treated blood [2000 U/L heparin (Heparin-Natrium-5000-ratiopharm)] was incubated with red blood cell lysis buffer [Thermo Fisher Scientific (eBioscience™)] prior to flow cytometric staining. Single cell suspensions for flow cytometric analysis were prepared from spleen, pLNs or thymus. Purified anti-CD16/CD32 antibodies [clone 93; Thermo Fisher Scientific (eBioscience™)] were used for blocking of Fc receptors in flow cytometric analysis. Fluorochrome-conjugated antibodies (clones) targeting following proteins were purchased from Thermo Fisher Scientific (eBioscience™): B220 (RA3-6B2), CD19 (eBio1D3), CD11b (M1/70), CD45 (30-F11), FOXP3 (FJK-16s), Gr-1 (RB6-8C5), IFN-γ (XMG1.2), NK1.1 (PK136), Ter119 (TER-119); from BD Biosciences: CD4 (GK1.5), CD8 (53-6.7), CD25 (PC61), CD62L (MEL14), CD69 (H1.2F3); or from Biolegend: CD3 (17A2), CD3e (145-2C11), CD44 (IM7), CD8.2b (53-5.8), IL-2 (JES6-5H4), KLRG1 (2F1), LY108 (330-AJ), PD1 (29F.1A12), TCR beta (H57-597), TNF-α (MP6-XT22). Total cell numbers were assessed by flow cytometry using counting beads Count Bright Beads (Invitrogen). Flow cytometry experiments were performed on a BD FACSCanto II, BD LSRFortessa (BD Bioscience) or Cytoflex (Beckman Coulter) and analyzed using BD FACSDiva V8.0 (BD Bioscience), CytExpert (Beckman Coulter) or FlowJo V10 (FlowJo, LLC) software. Intracellular stainings to analyze Ki67 protein levels were performed with the FITC Mouse Anti-Ki-67 Set (BD Pharmingen™) and the BD Cytofix/Cytoperm™ Fixation/Permeabilization Solution Kit (BD Bioscience) according to manufacturer’s instructions. Analysis of cytokine production was performed using the BD Cytofix/Cytoperm™ Fixation/Permeabilization Solution Kit (BD Bioscience) according to manufacturer’s instructions. Intracellular stainings for the analysis of Foxp3 levels were performed using the eBioscience™ Foxp3/Transcription Factor Staining Kit (Thermo Fisher Scientific) according to manufacturer’s instructions. Apoptosis staining was performed with the eBioscience™ Annexin V Apoptosis Detection Kit eFluor™ 450 (Thermo Fisher Scientific) according to manufacturer’s instructions. For staining of virus-specific CD8+ T cells upon LCMV infection, GP33 tetramers coupled to PE provided by the NIH Tetramer Core Facility were used. Tetramer staining was performed at 37°C prior to incubation with anti-CD16/32, followed by incubation with surface marker antibodies and eBioscience™ Fixable Viability Dye eFluor 780 (Thermo Fisher Scientific). Samples from LCMV infected mice were fixed in 4% Paraformaldehyde (Sigma) prior to flow cytometric analysis.

### Immunoblotting

Whole thymocyte lysates were prepared in 1x Laemmli buffer, incubated at 95°C for 5 min and sonicated for 15 min at room temperature. Protein concentrations were determined using Pierce™ BCA Protein Assay Kit (Thermo Fisher Scientific). Equal numbers of splenic CD8+ T cells were lysed in SDS-sample buffer [5% SDS (Biomol), 5% glycerol (Merck), 2.5% 2-mercaptoethanol and a trace amount of bromophenol blue sodium salt (Merck) in 375 mM Tris/HCl (pH 6.8)]. Proteins were separated by SDS/PAGE and transferred to a nitrocellulose membrane (Whatman^®^ Protran^®^). Membranes were blocked in 5% milk in pY-TBST buffer (10 mM Tris/HCl pH 7.4, 75 mM NaCl, 1 mM EDTA, 0.1% Tween-20) and probed with antibodies against HSC-70 (sc-7298) and CDK6 (sc-7180) overnight. Detection of bound antibodies was performed by incubation with horseradish peroxidase-conjugated anti-rabbit (CST, 7074S) or anti-mouse (CST, 7076S) antibodies followed by chemiluminescent detection using Clarity Western ECL substrate (BioRad) and the ChemiDocT XRS+ Molecular Imager (BioRad). Images were processed by Image Lab software (BioRad).

### Metabolic Extracellular Flux Analysis

Splenic naïve CD8+ T cells were cultured for 48h on αCD3/αCD28-coated wells in presence of IL-2. Cells were adjusted to 2.25*10^5^ cells per 180 µl of Agilent Seahorse XF RPMI medium (pH: 7.4, 5 mM Hepes). The medium was supplemented with 1 mM pyruvate, 2 mM glutamine and 10 mM glucose for Mito Stress Test or only with 2 mM glutamine for Glycolysis Stress Test. Per well, 180 µl containing 2.25*10^5^ cells were loaded onto a Seahorse XFe/XF96 cell culture microplate pre-coated with Cell Tak according to manufacturer’s instructions (Corning, 14.8 µg/ml) followed by centrifugation at 200 g for 2 min and subsequent resting for 45 min at 37°C in a non-CO2 incubator. Oxygen consumption rates (OCR) (pmol/min) and extracellular acidification rates (ECAR) (mpH/min) were measured using the Seahorse XF-96 metabolic extracellular flux analyzer (Agilent). Oligomycin, carbonyl cyanide p-trifluoro-methoxyphenyl hydrazone (FCCP), rotenone and antimycin A (Sigma) were prepared as 2.5 mM stock solutions in DMSO (Sigma, cell culture grade) and diluted accordingly in the respective Seahorse XF RPMI Medium prior to the assay. Glucose and 2-deoxy-glucose (Sigma) were dissolved on the day of the assay in Seahorse XF RPMI Medium (ph: 7.4, 5 mM Hepes, 2 mM glutamine). After 15 min of equilibration and measurement of basal respiration at 37°C inside the machine, cells were subjected for the Mito Stress Test to 2 µM oligomycin (Injection 1), after 15 min to 1 µM FCCP (Injection 2) and finally to 1 µM rotenone and antimycin A (Injection 3) for additional 15 min. During the Glycolysis Stress Test, cells were subjected to 25 mM glucose (Injection 1) for 15 min followed by 2 µM oligomycin for 15 min (Injection 2) and finally to 50 mM 2-deoxy-glucose (Injection 3). The assays were stopped 30 minutes after the last injection. Oxygen related to the mitochondrial ATP production was calculated from the drop in OCR after blocking mitochondrial ATP synthase with 2 µM oligomycin. Spare respiratory capacity (in %) is calculated as the maximal possible OCR after chemical uncoupling (1 µM FCCP) in relation to the basal OCR (first 15 minutes of the assay). Glycolysis is given as the ECAR change upon 25 mM glucose addition to glucose and pyruvate starved cells (1h), glycolytic reserve (%) is relative to the increase in ECAR after ATP synthase inhibition (2 µM oligomycin). Glycolytic capacity was calculated as the relation between the maximal possible ECAR (2 µM oligomycin) and the basal non glycolytic ECAR (ECAR after addition of 50 mM 2-deoxy glucose which blocks glycolysis). All values were normalized to the protein concentration per well determined by Pierce BCA assay (Thermo Fisher Scientific). Basal and maximal respiration, ATP production, glycolysis and glycolytic capacity were normalized to the corresponding average values of the *Cdk6* ^fl/fl^ control cells in the respective experiment.

### Redirected Cytotoxicity Assay

In the redirected killing assay, the FcγR+ murine mastocytoma cell line P815 was used as target (T) cells and was labelled with 1 µM carboxyfluorescein diacetate succinimidyl ester (CFSE, Molecular Probes, CellTrace CFSE Cell Proliferation Kit). CD8+ T cells after six days of culture were used as effector (E) cells. 5*10^4^ CFSE-labelled P815 cells were incubated with CD8+ T cells at different E:T ratios for 3h at 37°C in the presence of anti-CD3 (4 µg/ml) or anti-CD16/32 (4 µg/ml) as a control. Cells were stained with SYTOX™ Blue Dead Cell Stain (Thermo Fisher Scientific) and the specific target cell lysis was assessed by flow cytometry. Percentage of specific lysis was calculated as follows: [% Sytox+ CFSE+ cells after co‐incubation with effector cells] – [% Sytox+ CFSE+ cells without addition of effector cells (spontaneous lysis control)].

### MC38 *In Vivo* Tumor Model

Mice were injected subcutaneously with 10^6^ MC38 cells into both flanks and the tumor growth was monitored every other day. 16 days post injection the mice were sacrificed and the tumor weights were determined. For flow cytometric analysis of tumor-infiltrating T cells, tumors were cut into ~5 mm^2^ pieces and single cell suspensions were obtained using the gentleMACS™ *Octo Dissociator* (Miltenyi Biotec) with digestion buffer containing Collagenase D (1 mg/mL; Sigma Aldrich) and DNase I (20 mg/mL; Roche).

### RT-qPCR

0.5-1*10^6^ CD8+ T cells, isolated with the MagniSort Mouse CD8 Naïve T cell Enrichment Kit (Thermo Fisher Scientific), were kept unstimulated or stimulated with 10 U/ml IFN-β (Millipore or Sigma) for 2h either *ex vivo* or upon 48h of αCD3/αCD28-mediated activation. Cells were lysed in RNA-Solv^®^ Reagent (Omega Bio-tek) and RNA isolation was performed according to manufacturer’s instructions. RNA concentrations were measured using Nanodrop OneC (Thermo Fisher Scientific). 1 µg RNA was reverse transcribed into cDNA using the iScript™ cDNA Synthesis Kit (BioRad). The quantitative RT-PCR was performed on a C1000 Touch Thermal Cycler CFX96 Real Time System (BioRad) using SsoAdvanced™ Universal SYBR^®^ Green Supermix (BioRad). The following primers were used: Mx1 fwd: GACTACCACTGAGATGACCCAGC, rev: ATTTCCTCCCCAAATGTTTTCA; Mx2 fwd: CCAGTTCCTCTCAGTCCCAAGATT, rev: TACTGGATGATCAAGGGAACGTGG; Tap1 fwd: CTGGCAACCAGCTACGGGT, rev: TGAGAAAGAGGATGTGGTGGG; Ube2d2 fwd: AGGTCCTGTTGGAGATGATATGTT, rev: TTGGGAAATGAATTGTCAAGAAA. Socs1 and Socs3 primers were purchased from Qiagen (QuantiTect Primer Assays; GeneGlobe Id - QT02488983 and QT01059268). Expression of the genes of interest was calculated relative to expression of the house-keeping gene Ube2d2. Relative expression values were normalized to the corresponding unstimulated *Cdk6* ^fl/fl^ control samples.

### LCMV Virus Infection Model

LCMV strain Clone 13 was grown on BHK-21 cells (ATCC CCL-10) and titer was determined in a modified focus forming assay using Vero cells (ATCC CCL-81) (35). The same assay was also used to determine the virus titer in the blood of mice at day 8 post infection. Mice were intravenously infected with 2*10^6^ focus forming units (FFU) of LCMV strain Clone 13.

### Blood Chemistry

Blood was collected from LCMV infected mice in tubes coated with clotting factor (Sarstedt), serum was prepared by centrifugation at 10.000 rpm for 5 min at 4°C. Mouse serum was pre-diluted 1:8 in PBS and levels of alanine aminotransferase (ALT) were spectrophotometrically analyzed using a Cobas C311 Analyzer (Roche).

### Statistical Analysis

Unpaired or paired *t*-tests and one-way or two-way ANOVA with Tukey’s *post hoc* tests were performed using GraphPad Prism version 5.00 (GraphPad Software). The level of significance is indicated for each experiment (*p < 0.05; **p < 0.01; ***p < 0.001).

## Results

### Loss of CDK6 or Its Kinase Activity Impairs *In Vitro* Expansion of CD8+ T Cells

**Figure 1:**
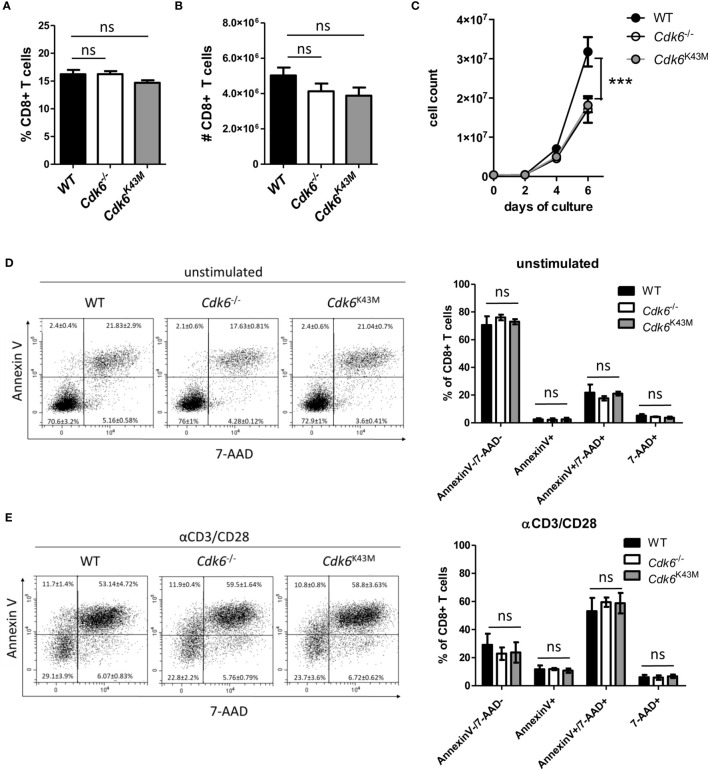
Loss of CDK6 or its kinase activity impairs *in vitro* expansion of CD8+ T cells. **(A, B)** Percentage **(A)** and total numbers **(B)** of CD3+CD8+ T cells were analyzed by flow cytometry in the spleen of WT, *Cdk6^-^* ^/-^ and *Cdk6* ^K43M^ mice (n=8-10). **(C)** Splenic CD8+ T cells were isolated, activated with αCD3/αCD28 for 48h and cultured in the presence of IL-2. Numbers of CD8+ T cells were analyzed every two days during culture (n=4). **(D, E)** Cultured CD8+ T cells were kept unstimulated **(D)** or re-stimulated with αCD3/αCD28 **(E)** for 24h (n=4). Apoptosis was analyzed by flow cytometric AnnexinV/7-AAD staining and representative dot blots are shown. **(A, B, D, E)** Bar graphs represent mean ± SEM, pooled from 2-3 independent experiments. **(C)** Symbols in growth curves represent mean ± SEM, pooled from two independent experiments; ***p < 0.001 (for comparison on d6), two-way ANOVA. ns, not significant.

Loss of CDK6 or its kinase activity in CDK6-deficient (*Cdk6* ^-/-^) and kinase-dead mutant CDK6 (*Cdk6* ^K43M^) knock-in mice is associated with thymic changes (5–7). This prompted us to investigate the frequency and absolute numbers of CD8+ T cells in the spleen of *Cdk6* ^-/-^ and *Cdk6* ^K43M^ mice. No significant differences were observed compared to wild-type (WT) controls (**Figures 1A, B**), indicating that CDK6 deficiency does not interfere with splenic CD8+ T cell abundance. While CDK6 deficiency is well tolerated under homeostatic conditions, phenotypes become evident under stimulation and stress conditions (9). Expansion of *Cdk6* ^-/-^ and *Cdk6* ^K43M^ CD8+ T cells was impaired upon αCD3/αCD28 stimulation *in vitro* compared to WT controls (**Figure 1C**). CDK6 has also been implicated in the regulation of cell survival, where the potential of CDK4/6 inhibitors to induce apoptosis depends on the cellular context (6, 10, 12, 13, 36–40). To test whether the impaired *in vitro* growth of *Cdk6* ^-/-^ and *Cdk6* ^K43M^ CD8+ T cells is paralleled by an increased sensitivity to cell death, we analyzed activation-induced cell death (AICD) of CD8+ T cells after four days of culture by re-stimulation with αCD3/αCD28 for 24h. No significant differences in the apoptotic response of CD8+ T cells were observed irrespective of the genotypes (**Figures 1D, E**). These data determine a role of CDK6 kinase activity in mitogen-induced CD8+ T cell expansion *in vitro*, while it is dispensable for the regulation of AICD.

### Loss of CDK6 Causes Transcriptional Deregulation of Metabolic Pathways and Interferon Signaling in CD8+ T Cells

**Figure 2:**
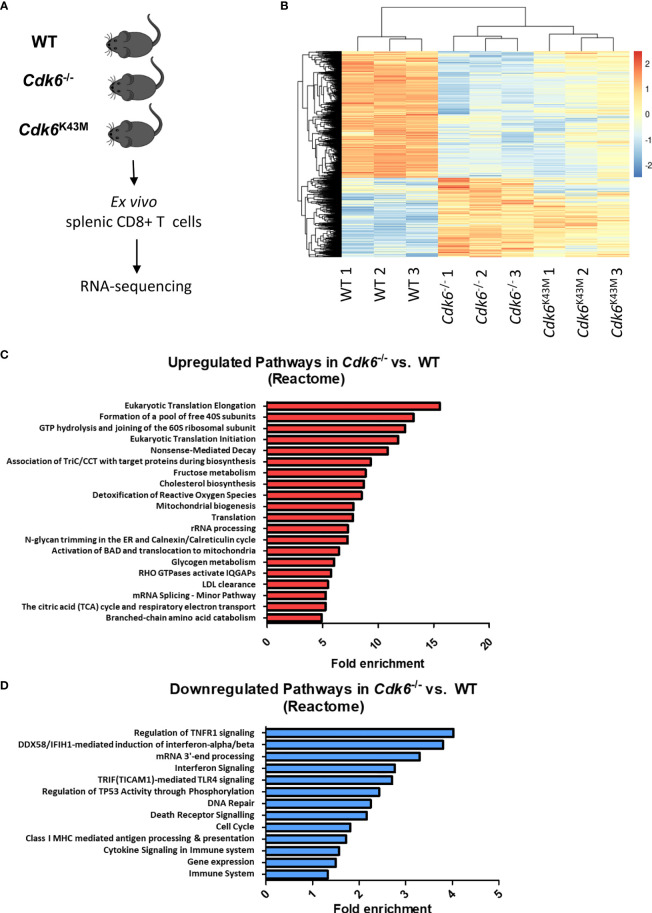
Loss of CDK6 deregulates major cellular processes, including metabolic pathways and cytokine signaling in CD8+ T cells. **(A)** Splenic CD3+CD8+ T cells were sorted *ex vivo* from WT, *Cdk6^-^* ^/-^ and *Cdk6* ^K43M^ mice and lysed for RNA-sequencing. Three biological replicates (each pooled from two mice) were prepared per genotype. **(B)** The heatmap depicts genes significantly deregulated (FDR < 0.05, >1.25x fold change) in *Cdk6^-^* ^/-^ *vs.* WT and *Cdk6* ^K43M^ *vs.* WT CD8+ T cells. **(C, D)** Pathway analysis was performed using the Reactome Pathway tool with genes that were significantly upregulated **(C)** or downregulated **(D)** in *Cdk6^-^* ^/-^ compared to WT CD8+ T cells. Significant pathways (FDR<0.05) are ranked by fold enrichment. Bar graphs show the top 20 upregulated non-redundant Reactome pathways in **(C)**, while all downregulated significant non-redundant pathways are depicted in **(D)**.

As CDK6 is not only a cell cycle kinase, but also acts as a transcriptional regulator (3, 4), we performed RNA-sequencing of *ex vivo* sorted splenic WT, *Cdk6* ^-/-^ and *Cdk6* ^K43M^ CD8+ T cells (**Figure 2A**). Under steady-state conditions, we detected 1503 upregulated genes in *Cdk6* ^-/-^ CD8+ T cells, including 672 genes that were also upregulated in *Cdk6* ^K43M^ CD8+ T cells. 2375 genes were downregulated in *Cdk6* ^-/-^ CD8 T cells, including 1680 genes that were also downregulated in *Cdk6* ^K43M^ CD8+ T cells (**Figure 2B**, **Figures S1A, B**). The functional consequences of these genes were probed by performing overrepresentation analysis with Reactome Pathways. The top 20 enriched pathways upregulated in *Cdk6* ^-/-^ compared to WT CD8+ T cells included cellular processes associated with translation and metabolic functions (**Figure 2C**) and overlapped with alterations found in *Cdk6* ^K43M^ CD8+ T cells, indicating a kinase-dependent regulation (**Figure S1C**). In line, CDK6 has previously been reported to regulate tumor cell metabolism in a kinase-dependent manner (38, 41). Pathways downregulated in *Cdk6* ^-/-^ CD8+ T cells included cell cycle, DNA repair and cytokine signaling in accordance with previously reported roles of CDK6 (12, 15, 24, 25, 40). Cell cycle and DNA repair were also found altered in *Cdk6* ^K43M^ CD8+ T cells (**Figure S1D**). Pathway analysis also revealed interferon (IFN) signaling as downregulated in *Cdk6* ^-/-^ CD8+ T cells. Type I IFN signaling plays an important role in anti-viral and anti-tumor responses of CD8+ T cells (42–44). To study deregulation of interferon-stimulated genes (ISGs) upon loss of CDK6 in more depth, we analyzed the overlap of the genes downregulated in *Cdk6* ^-/-^ CD8+ T cells with a set of core type I and type II ISGs, defined based on the interferome database (see *Materials and Methods*). This strategy identified a total of 21 type I ISGs, 9 type II ISGs and 12 non-specific ISGs downregulated in *Cdk6* ^-/-^ CD8+ T cells (**Figure S1E**). Although the interferon signaling pathway was not among the significantly downregulated pathways in *Cdk6* ^K43M^ CD8+ T cells, half of the ISGs downregulated in *Cdk6* ^-/-^ CD8+ T cells were also downregulated in *Cdk6* ^K43M^ CD8+ T cells (**Figure S1E**, marked in blue). This indicates that ISG regulation partially requires CDK6 kinase activity. Altogether, loss of CDK6 and its kinase activity are associated with global changes in the transcriptome of steady-state CD8+ T cells, including changes in metabolic activity and interferon signaling.

### *Cdk6* ^fl/fl^ CD4-Cre Mice Do Not Have a Defect in Thymic T Cell Development and Splenic CD8+ T Cell Numbers, While *In Vitro* Expansion of CD8+ T Cells Is Impaired

**Figure 3:**
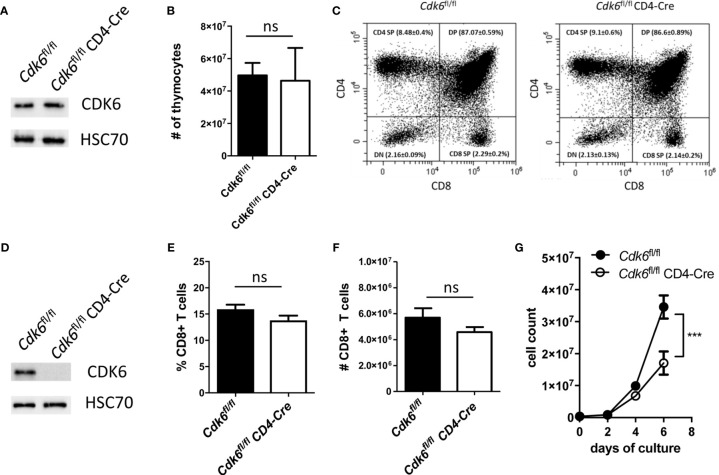
*Cdk6^fl/fl^* CD4-Cre mice do not show a defect in thymic T cell development and splenic CD8+ T cell numbers, while *in vitro* expansion of CD8+ T cells is impaired. **(A)** Thymocytes were lysed from *Cdk6* ^fl/fl^ and *Cdk6* ^fl/fl^ CD4-Cre mice for western blot analysis of CDK6 protein levels. HSC70 served as a loading control. **(B)** Absolute numbers of thymocytes were analyzed in *Cdk6* ^fl/fl^ and *Cdk6* ^fl/fl^ CD4-Cre mice (n=4-7). **(C)** The frequencies of CD4/CD8 double-negative (DN), CD4/CD8 double-positive (DP), CD4 single-positive (CD4 SP) and CD8 single-positive (CD8 SP) thymocytes were analyzed by flow cytometry in *Cdk6* ^fl/fl^ CD4-Cre mice and *Cdk6* ^fl/fl^ controls (n=4-7). Representative dot plots are shown. **Figure S2A** shows the corresponding bar graph with mean ± SEM values. **(D)** Splenic CD8+ T cells from *Cdk6* ^fl/fl^ and *Cdk6* ^fl/fl^ CD4-Cre mice were cultured for six days and lysed for western blot analysis of CDK6 protein levels. HSC70 served as a loading control. **(E, F)** Percentage **(E)** and total numbers **(F)** of splenic CD3+CD8+ T cells were analyzed by flow cytometry in *Cdk6* ^fl/fl^ and *Cdk6* ^fl/fl^ CD4-Cre mice (n=7-9). **(G)** Splenic CD8+ T cells were isolated from *Cdk6* ^fl/fl^ and *Cdk6* ^fl/fl^ CD4-Cre mice, activated with αCD3/αCD28 for 48h and cultured in the presence of IL-2. Numbers of CD8+ T cells were analyzed every two days during the culture (n=4). **(B, E, F)** Bar graphs represent mean ± SEM, pooled from 2-3 independent experiments. **(G)** Symbols in growth curves represent mean ± SEM, pooled from two independent experiments; ***p < 0.001 (for comparison on d6), two-way ANOVA. ns, not significant.

*Cdk6* ^-/-^ and *Cdk6* ^K43M^ mice allow to decipher kinase-dependent and -independent functions of CDK6. The majority of the top deregulated pathways depend on CDK6 kinase activity. It remains however unclear, whether the effects observed in CD8+ T cells are cell-intrinsic or caused by changes in other cell types. To address that, we generated mice with a T cell-specific deletion of CDK6 by crossing *Cdk6* ^fl/fl^ (29) to CD4-Cre mice (30). In this mouse model floxed genes are deleted at the CD4/CD8 double-positive (DP) thymocyte stage and all T cell populations are affected (45). Therefore, this model allows to investigate, whether the global effects of CDK6 deficiency on CD8+ T cells are re-capitulated upon T cell-intrinsic loss of CDK6. Of note, we failed to induce an efficient CDK6 deletion in *Cdk6* ^fl/fl^ CD4-Cre whole thymocytes (**Figure 3A**) and consequently no changes in thymocyte numbers or altered frequencies of the different thymocyte populations were detected (**Figures 3B, C**, **Figure S2A**). In contrast, peripheral splenic CD8+ T cells efficiently deleted CDK6 (**Figure 3D**), which was not paralleled by significant differences in the percentage and absolute numbers of CD8+ T cells in the spleen (**Figures 3E, F**). Similar to *Cdk6* ^-/-^ and *Cdk6* ^K43M^, isolated naïve *Cdk6* ^fl/fl^ CD4-Cre CD8+ T cells showed an expansion defect *in vitro* upon αCD3/αCD28 stimulation (**Figure 3G**). This is most likely caused by a delayed proliferation as indicated by decreased CFSE proliferation dye dilution in cultured *Cdk6* ^fl/fl^ CD4-Cre compared to *Cdk6* ^fl/fl^ CD8+ T cells (**Figure S2B, C**). Taken together, *Cdk6* ^fl/fl^ CD4-Cre mice circumvent a thymic developmental defect, while peripheral CD8+ T cells show a proliferation defect *in vitro* similar to those isolated from *Cdk6* ^-/-^ and *Cdk6* ^K43M^ mice. This led us to conclude that *Cdk6* ^fl/fl^ CD4-Cre mice are a useful model to study the functional consequences of CDK6 loss in peripheral T cells.

### Loss of CDK6 Increases Mitochondrial Respiration of CD8+ T Cells

**Figure 4:**
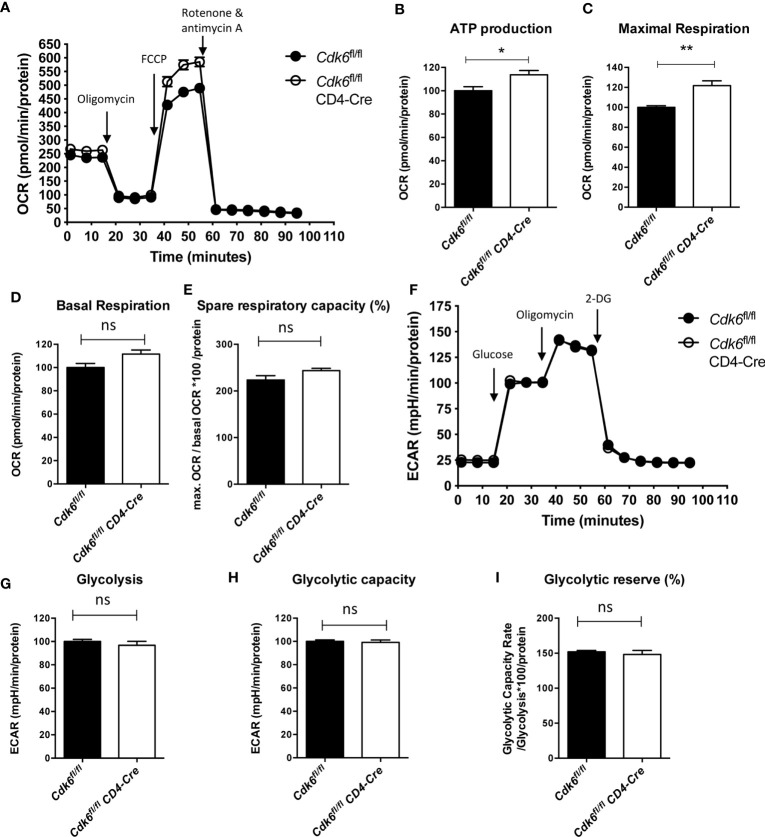
Loss of CDK6 increases mitochondrial respiration of CD8+ T cells *in vitro*. Naïve CD8+ T cells were isolated from spleens of *Cdk6^fl^* ^/fl^ and *Cdk6^fl^* ^/fl^ CD4-Cre mice and cultured for 48h on αCD3/αCD28 coated wells in presence of IL-2. **(A–E)** Agilent Seahorse XF Cell Mito Stress Test was performed with cultured CD8+ T cells. **(A)** Oxygen consumption rate (OCR) curves are shown. Injections of oligomycin (Injection 1), FCCP (Injection 2) and rotenone and antimycin A (Injection 3) are indicated by arrows. ATP production **(B)**, maximal respiration **(C)**, basal respiration **(D)** and spare respiratory capacity (as %) **(E)** were calculated for *Cdk6* ^fl/fl^ and *Cdk6* ^fl/fl^ CD4-Cre CD8+ T cells (n=4). **(F–I)** Agilent Seahorse XF Glycolysis Stress Test was performed with cultured CD8+ T cells. **(F)** Extracellular acidification rate (ECAR) curves are shown. Injections of glucose (Injection 1), oligomycin (Injection 2) and 2-deoxy-glucose (2-DG) (Injection 3) are indicated by arrows. Glycolysis **(G)**, glycolytic capacity **(H)** and glycolytic reserve (as %) **(I)** were calculated for *Cdk6* ^fl/fl^ and *Cdk6* ^fl/fl^ CD4-Cre CD8+ T cells (n=4). **(B–E, G–I)** Bar graphs represent mean ± SEM, pooled from two independent experiments. **p < 0.01, *p < 0.05, unpaired t-test. **(A, F)** Symbols in curves represent mean ± SEM (n=4 biological replicates per genotype with 8 technical replicates). ns, not significant.

Pathways related to metabolism, including “TCA cycle and respiratory electron transport” and “Mitochondrial biogenesis”, were upregulated upon loss of CDK6 or its kinase activity (**Figure 2C**, **Figure S1C**). Metabolic activities of T cells are linked to their functionality (46). We thus performed an Agilent Seahorse XF Cell Mito Stress Test to analyze the mitochondrial respiration in αCD3/αCD28-activated *Cdk6* ^fl/fl^ CD4-Cre CD8+ T cells *in vitro* (**Figure 4A**). We found an enhanced maximal respiration as well as mitochondrial ATP production in *Cdk6* ^fl/fl^ CD4-Cre CD8+ T cells compared to *Cdk6* ^fl/fl^ controls (**Figures 4B, C**). We also observed a trend for increased basal respiration (p = 0.06) and spare respiratory capacity (SRC) (p = 0.1) in *Cdk6* ^fl/fl^ CD4-Cre CD8+ T cells (**Figures 4D, E**). SRC is a measure for the extra mitochondrial capacity available for increased energy demand (47, 48). To investigate potential differences in glycolysis, we performed an Agilent Seahorse XF Glycolysis Stress Test, but failed to detect significant differences in the glycolytic activity of *Cdk6* ^fl/fl^ CD4-Cre CD8+ T cells compared to *Cdk6* ^fl/fl^ controls (**Figures 4F–I**). As indicated by RNA-seq in steady-state *Cdk6* ^-/-^ and *Cdk6* ^K43M^ CD8+ T cells, T cell-intrinsic CDK6 represses the mitochondrial respiration capacity of CD8+ T cells.

### T Cell-Intrinsic Loss of CDK6 Does Not Affect MC38 Tumor Growth *In Vivo*

**Figure 5:**
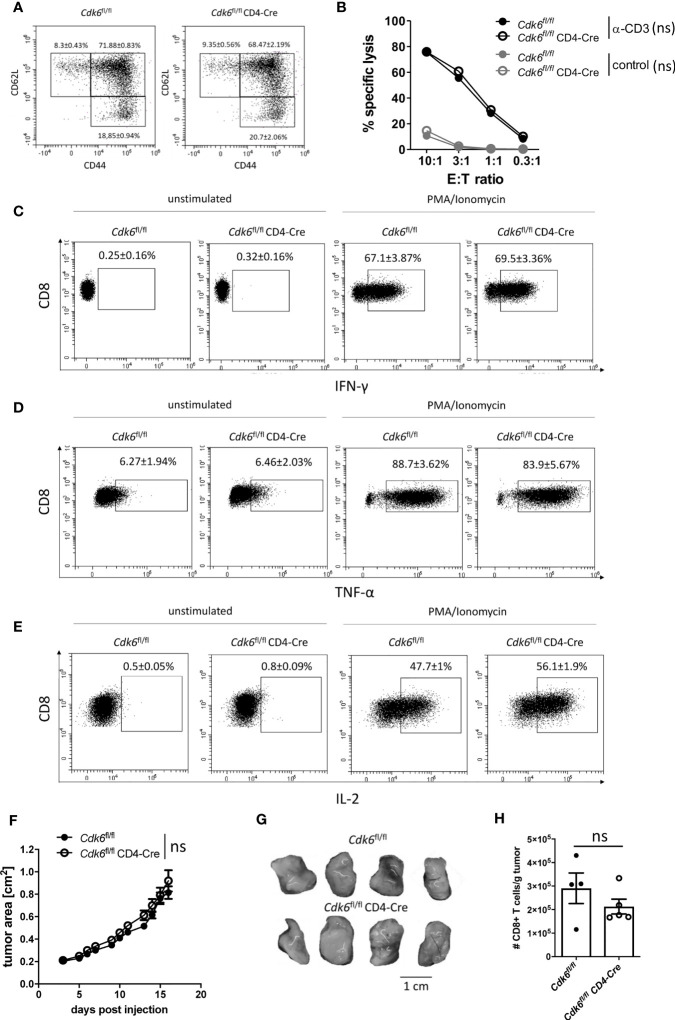
T cell-intrinsic loss of CDK6 does not affect MC38 tumor growth *in vivo*. ***(*A–E)** Splenic CD8+ T cells were isolated from *Cdk6* ^fl/fl^ and *Cdk6* ^fl/fl^ CD4-Cre mice, activated with αCD3/αCD28 for 48h and cultured in the presence of IL-2. **(A)** Percentages of naïve (CD62L+CD44-), central memory (CD62L+CD44+) and effector memory (CD62L-CD44+) CD8+ T cells were analyzed after six days of culture by flow cytometry (n=4). Representative dot plots are shown. **Figure S3A** shows the corresponding bar graph with mean ± SEM values, pooled from two independent experiments. **(B)** Redirected cytotoxicity assay with the P815 target (T) cell line was performed with *Cdk6* ^fl/fl^ and *Cdk6* ^fl/fl^ CD4-Cre CD8+ T cells six days upon culture as effector (E) cells. Cells were incubated at different E:T ratios for 3h in the presence of αCD3 or with an FcR-blocking reagent (control). Specific lysis of CFSE-labeled target cells was analyzed by flow cytometry (n=2). **(C–E)** Cultured *Cdk6* ^fl/fl^ and *Cdk6* ^fl/fl^ CD4-Cre CD8+ T cells were kept unstimulated or stimulated with PMA/Ionomycin for 4h and percentages of IFN-γ **(C)**, TNF-α **(D)** and IL-2 **(E)** positive CD8+ T cells were analyzed by intracellular flow cytometric staining (n=4-5). Representative dot plots are shown. **Figure S3E–G** show the corresponding bar graphs with mean ± SEM values, pooled from two independent experiments. **(F–H)** *Cdk6* ^fl/fl^ and *Cdk6* ^fl/fl^ CD4-Cre mice were injected subcutaneously into both flanks with 10^6^ MC38 tumor cells. **(F)** Growth of the tumors was monitored by assessment of tumor area every 1-2 days. **(G)** Representative tumor pictures are shown. **Figure S3H** shows the bar graph with the corresponding tumor weights (n=4-5 mice per genotype (8-10 tumors per genotype)). **(H)** Absolute numbers (normalized to tumor weight) of tumor-infiltrating CD8+ T cells were analyzed by flow cytometry (n=4-5). **(B, F)** Symbols represent mean ± SEM from one experiment. **(H)** Bar graph represents mean ± SEM with symbols representing values from individual tumors from one experiment. ns, not significant.

Mitochondrial metabolism plays a role in T cell activation and memory formation (46, 49). Furthermore, mitochondrial biogenesis and the mitochondrial SRC were described as indicators of CD8+ T cell effector function (48). After 48h of αCD3/αCD28-mediated activation of naïve CD8+ T cells, no significant differences in the expression levels of the activation marker CD69, CD44 and the proliferation marker Ki67 were detected. In contrast, we found a minor decrease in CD25 (IL-2Rα) and an increase in CD62L surface levels on *Cdk6* ^fl/fl^ CD4-Cre CD8+ T cells (**Figures S2D, E**). The difference in CD62L expression did not result in significant differences in the proportion of *Cdk6* ^fl/fl^ CD4-Cre CD8+ T cells with a naïve (CD62L+CD44-), central memory (CD62L+CD44+) or effector memory (CD62L-CD44+) phenotype compared to *Cdk6* ^fl/fl^ controls upon six days of culture (**Figure 5A**, **Figure S3A**). Under steady-state conditions *Cdk6* ^fl/fl^ CD4-Cre mice displayed an increased proportion of naïve (CD62L+CD44-) and decreased proportion of central memory (CD62L+CD44+) splenic CD8+ T cells compared to *Cdk6* ^fl/fl^ mice (**Figure S3B**). This indicates that upon *in vitro* expansion isolated naïve CDK6-deficient CD8+ T cells overcome the differences observed *ex vivo*, acquiring a central memory phenotype similar to *Cdk6* ^fl/fl^ controls. Similar to activated CD8+ T cells no differences in the expression of CD69 and Ki67 were detected *ex vivo* between genotypes (**Figure S3C, D**).

We observed no significant differences in the cytotoxic activity and in the production of the effector cytokines IFN-γ and TNF-α between cultured *Cdk6* ^fl/fl^ CD4-Cre CD8+ T cells and *Cdk6* ^fl/fl^ controls (**Figures 5B–D**, **Figures S3E, F**). In contrast, *Cdk6* ^fl/fl^ CD4-Cre CD8+ T cells showed increased IL-2 production compared to *Cdk6* ^fl/fl^ CD8+ T cells (**Figure 5E**, **Figure S3G**). Taken together, T cell-intrinsic loss of CDK6 diminishes *in vitro* expansion and enhances mitochondrial respiration, while the absence of CDK6 has no major impact on CD8+ T cell effector function *in vitro*.

CDK4/6 inhibitor treatment increases T cell-mediated anti-tumor responses, involving T cell-extrinsic and -intrinsic mechanisms (24, 25, 27). To see whether enhanced anti-tumor immunity *in vivo* is related to the absence of CDK6 in T cells, we subcutaneously injected *Cdk6* ^fl/fl^ CD4-Cre and *Cdk6* ^fl/fl^ mice with the colon carcinoma cell line MC38, which is surveilled by CD8+ T cells (50–52). We did not observe significant differences in the tumor growth and tumor weights between *Cdk6* ^fl/fl^ CD4-Cre and *Cdk6* ^fl/fl^ mice (**Figures 5F, G**, **Figure S3H**). Furthermore, the absolute numbers of tumor-infiltrating CD8+ T cells in MC38-inflicted tumors did not significantly differ between *Cdk6* ^fl/fl^ CD4-Cre and *Cdk6* ^fl/fl^ mice (**Figure 5H**). These data indicate that T cell-intrinsic CDK6 expression does not have an impact on tumor surveillance *in vivo*.

### T Cell-Intrinsic Loss of CDK6 Reduces Numbers of MC38 Tumor-Infiltrating CD4+ T Cells

As the CD4-Cre model deletes in all peripheral T cell subsets (45), the *Cdk6* ^fl/fl^ CD4-Cre mouse model does not dissect CD8+ T cell-intrinsic roles of CDK6 *in vivo.* Thus, we cannot exclude that the absence of any difference in anti-tumor responses *in vivo* is related to a complex interplay between phenotypes of different T cell subsets that compensate each other. To gain insights into effects of CDK6 loss on non-CD8+ T cell populations, we characterized CD4+ T cell subsets. No significant differences in the numbers and frequencies of total CD4+ T cells as well as Tregs (CD25+Foxp3+CD4+ T cells) were observed in spleens and peripheral lymph nodes (pLNs) when comparing *Cdk6* ^fl/fl^ and *Cdk6* ^fl/fl^ CD4-Cre mice (**Figures S4A–D**). We found an increased proportion of naïve (CD62L+CD44-) and decreased proportion of effector memory (CD62L-CD44+) CD4+ T cells in spleens and pLNs of *Cdk6* ^fl/fl^ CD4-Cre compared to *Cdk6* ^fl/fl^ mice. A significantly decreased proportion of central memory (CD62L+CD44+) CD4+ T cells was only observed in pLNs (**Figures S4E, F**). As CDK6-deficient CD8+ T cells produce more IL-2, we explored IL-2 production in cultured CD4+ T cells. Upon 72h of αCD3/αCD28-mediated activation, IL-2 production was higher in *Cdk6* ^fl/fl^ CD4-Cre CD4+ T cells compared to *Cdk6* ^fl/fl^ controls (**Figures S5A, B**). The expression of CD25 was comparable, whereas CD69 expression was higher in cultured *Cdk6* ^fl/fl^ CD4-Cre CD4+ T cells (**Figures S5C–F**). The analysis of MC38 tumor-infiltrating CD4+ T cells revealed a decreased number of CD4+ T cells in tumors from *Cdk6* ^fl/fl^ CD4-Cre mice (**Figure S6A**). This decrease also affected Treg numbers (**Figure S6B**). Tregs from *Cdk6* ^fl/fl^ CD4-Cre mice showed higher expression levels of the activation marker CD69, while the increase on total CD4+ T cells did not reach statistical significance (**Figures S6C, D**). No differences were observed in the percentages of CD62L+CD44-, CD62L+CD44+ and CD62L-CD44+ CD4+ T cells and Tregs between genotypes (**Figures S6E, F**). Our data indicate that T cell-intrinsic loss of CDK6 results in higher proportions of naïve CD8+ and CD4+ T cells under steady-state conditions. Moreover, CDK6-deficiency enhances IL-2 production in activated CD8+ and CD4+ T cells. *In vivo* T cell-intrinsic loss of CDK6 reduces numbers of tumor-infiltrating CD4+ T cells and Tregs without affecting tumor growth.

### T Cell-Intrinsic Loss of CDK6 Impairs Type I IFN Response *In Vitro*, but Does Not Affect Response to LCMV Infection *In Vivo*

**Figure 6:**
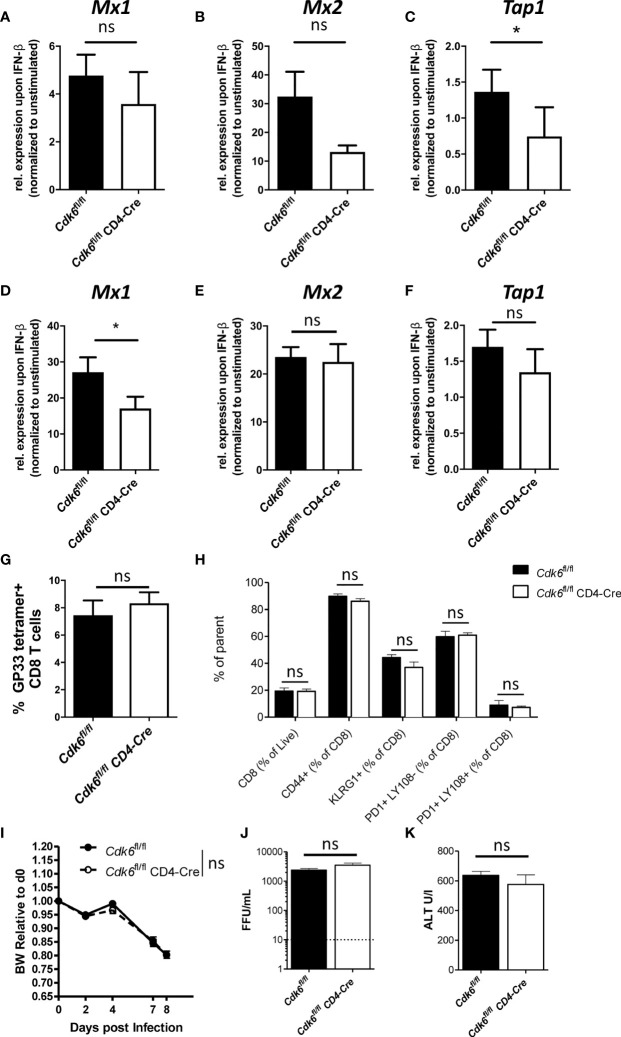
T cell-intrinsic loss of CDK6 partially impairs type I IFN response, but does not affect response to LCMV Clone 13 *in vivo*. **(A–C)** Splenic CD8+ T cells were isolated from *Cdk6* ^fl/fl^ and *Cdk6* ^fl/fl^ CD4-Cre mice and stimulated with 10U/ml IFN-β for 2h. Cells were lysed and relative expression levels (normalized to *Ube2d2* as a house-keeping gene) of *Mx1* **(A)**, *Mx2* **(B)** and *Tap1* **(C)** were analyzed by qPCR. Fold of induction of genes over unstimulated *Cdk6* ^fl/fl^ controls are shown (n=3). **(D–F)** Splenic CD8+ T cells were isolated from *Cdk6* ^fl/fl^ and *Cdk6* ^fl/fl^ CD4-Cre mice and activated with αCD3/αCD28 for 48h in the presence of IL-2. Cultured CD8+ T cells were stimulated with 10U/ml IFN-β for 2h. Cells were lysed and relative expression levels (normalized to *Ube2d2* as a house-keeping gene) of *Mx1* **(D)**, *Mx2* **(E)** and *Tap1* **(F)** were analyzed by qPCR. Fold of induction of genes over unstimulated *Cdk6* ^fl/fl^ controls are shown (n=3-4). **(G–K)** *Cdk6* ^fl/fl^ and *Cdk6* ^fl/fl^ CD4-Cre mice were intravenously infected with 2*10^6^ focus forming units (FFU) of LCMV Clone 13 and CD8+ T cells were analyzed in the blood 8 days post infection by flow cytometry (n=5). **(G)** Percentage of GP33-tetramer positive CD8+ T cells are shown. **(H)** Frequency of CD8+ T cells, CD44+, KLRG1+, PD1+Ly108-, PD1+Ly108+ CD8+ T cells were analyzed. **(I)** Body weight (BW) of LCMV infected *Cdk6* ^fl/fl^ and *Cdk6* ^fl/fl^ CD4-Cre mice was monitored and is shown relative to BW on d0. Virus load **(J)** and ALT levels **(K)** in the blood of LCMV infected *Cdk6* ^fl/fl^ and *Cdk6* ^fl/fl^ CD4-Cre mice were measured on day 8 and 11 post infection, respectively. **(A–F)** Bar graphs represent mean ± SEM, pooled from 2-3 independent experiments. *p < 0.05, paired t-test. **(G–K)** Bar graphs or symbols represent mean ± SEM from one experiment. ns, not significant.

ISGs are down-regulated in *Cdk6* ^-/-^ and *Cdk6* ^K43M^ CD8+ T cells under steady-state conditions (**Figure S1E**). To validate this finding, we analyzed expression of three ISGs (*Mx1, Mx2* and *Tap1*) upon short term *ex vivo* IFN-β stimulation of naïve *Cdk6* ^fl/fl^ and *Cdk6* ^fl/fl^ CD4-Cre CD8+ T cells. While we observed a significantly decreased expression of Tap1 in *Cdk6* ^fl/fl^ CD4-Cre CD8+ T cells, we observed a tendency for decreased levels of *Mx1* (p = 0.14) and *Mx2* (p = 0.17) expression upon IFN-β stimulation for 2h, however not reaching statistical significance (**Figures 6A–C**). IFN signaling has been shown to depend on the T cell activation state (53). Therefore, we activated *Cdk6* ^fl/fl^ and *Cdk6* ^fl/fl^ CD4-Cre CD8+ T cells with αCD3/αCD28 in presence of IL-2 for 48h and subsequently repeated the IFN-β stimulation for 2h. In the activated state, the changes regarding a decreased expression of *Mx1* reached significance, while we observed only a tendency for decreased levels of *Tap1* (p = 0.076) upon IFN-β stimulation. The levels of *Mx2* were comparable under these conditions between genotypes (**Figures 6D–F**). These data led us to conclude that T cell-intrinsic loss of CDK6 is associated with a weakened response to type I IFN stimulation.

IFN signaling plays an important role during anti-viral responses against LCMV infection (43, 54–59). In chronic LCMV infection, IFN signaling is important for activation and expansion of CD8+ T cells (43), but also drives T cell exhaustion (60). As CDK6 interferes with type I IFN signaling in CD8+ T cells, we explored CD8+ T cell responses to LCMV infection and infected *Cdk6* ^fl/fl^ CD4-Cre mice and *Cdk6* ^fl/fl^ controls with LCMV Clone 13. At day 8 post infection we did not observe any significant difference in the percentage of antigen-specific (GP33-tetramer positive) CD8+ T cells (**Figure 6G**). The proportion of CD8+ T cells expressing the activation marker CD44 and the differentiation marker KLRG1, indicative of effector-like T cells (61), were also unchanged between *Cdk6* ^fl/fl^ CD4-Cre and *Cdk6* ^fl/fl^ mice (**Figure 6H**). To look for T cell exhaustion, we made use of the markers PD-1 and Ly108, which is a surrogate marker for TCF-1, a key transcription factor of progenitor exhausted CD8+ T cells (61). T cell-intrinsic type I IFN signaling was previously linked to reduced numbers of TCF1-dependent less exhausted progenitor CD8+ T cells in LCMV Clone 13 infection, leading to a less efficiently controlled virus infection (60). Again, T cell-intrinsic loss of CDK6 did not affect the proportion of Ly108+PD1+ progenitor exhausted as well as exhausted Ly108-PD1+ CD8+ T cells in infected *Cdk6* ^fl/fl^ CD4-Cre compared to *Cdk6* ^fl/fl^ mice (**Figure 6H**). Furthermore, no difference in infection-associated weight loss, which depends on CD8+ T cell-intrinsic type I IFN signaling (43), occurred (**Figure 6I**). Additionally, the virus load and levels of the liver damage marker ALT were unaltered between genotypes (**Figures 6J, K**). These data indicate that T cell-intrinsic CDK6 regulates IFN signaling, but is dispensable for the *in vivo* CD8+ T cell response to LCMV Clone 13 infection.

### CDK6 Regulates Negative Feedback Loops of Type I IFN Responses

**Figure 7:**
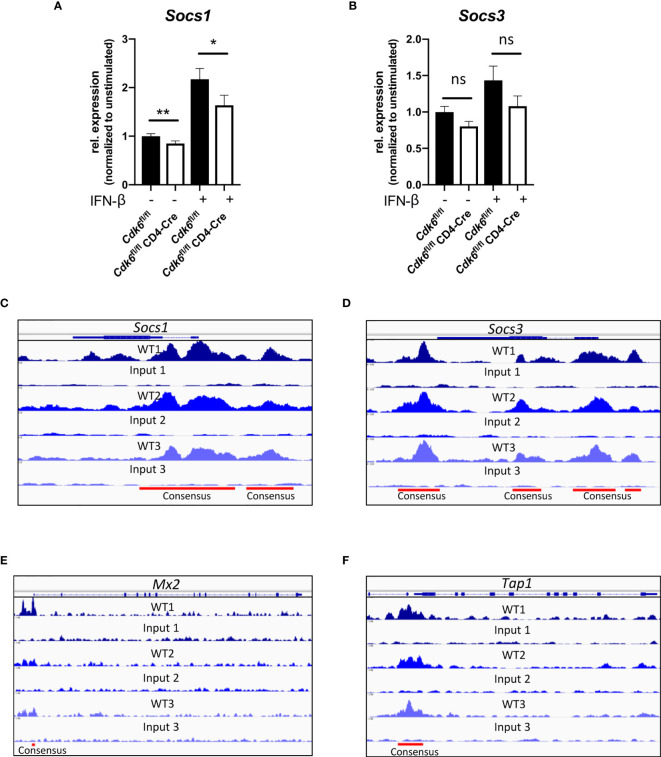
CDK6 regulates negative feedback loops of type I IFN signaling. **(A, B)** Splenic CD8+ T cells were isolated from *Cdk6* ^fl/fl^ and *Cdk6* ^fl/fl^ CD4-Cre mice and activated with αCD3/αCD28 for 48h in the presence of IL-2. Cultured CD8+ T cells were kept unstimulated or stimulated with 10U/ml IFN-β for 2h. Cells were lysed and relative expression levels (normalized to *Ube2d2* as a house-keeping gene) of *Socs1* **(A)** and *Socs3* **(B)** were analyzed by qPCR. Relative expression values are shown normalized to unstimulated *Cdk6* ^fl/fl^ controls (n=6). **(C–F)** CDK6 ChIP-seq was performed in p185^BCR-ABL^-transformed B cell lines (12). CDK6 ChIP-seq peaks at the *Socs1* **(C)**, *Socs3* **(D)**, *Mx2* **(E)** and *Tap1* **(F)** loci are shown. Input samples are shown as controls. Consensus peaks are indicated with lines. **(A, B)** Bar graphs represent mean ± SEM, pooled from three independent experiments, **p < 0.01, *p < 0.05, paired t-test. ns, not significant.

We wondered what caused the discrepancy between the impaired IFN signaling and reduced T cell expansion and the absence of any effects upon virus or tumor challenge *in vivo* upon T cell-intrinsic loss of CDK6. One potential explanation might be that negative regulators of IFN signaling are also affected by the absence of CDK6, providing a compensatory effect in long-term experiments. According to our RNA-seq data negative regulators of IFN responses, namely the Suppressor of cytokine signaling (SOCS) family members *Socs1* and *Socs3* (62–66), were downregulated in CD8+ T cells upon loss of CDK6 (**Figure S1E**). We detected decreased levels of *Socs1* expression in αCD3/αCD28-activated *Cdk6* ^fl/fl^ CD4-Cre compared to *Cdk6* ^fl/fl^ CD8+ T cells (**Figure 7A**). Upon IFN-β stimulation, *Socs1* expression was upregulated to a lower extent in *Cdk6* ^fl/fl^ CD4-Cre compared to *Cdk6* ^fl/fl^ CD8+ T cells (**Figure 7A**). We also observed a tendency for reduced levels of *Socs3* (p = 0.07) in unstimulated *Cdk6* ^fl/fl^ CD4-Cre compared to *Cdk6* ^fl/fl^ CD8+ T cells. However, statistical significance was not reached for this difference as well as for a difference in IFN-β-induced *Socs3* (p = 0.137) levels between *Cdk6* ^fl/fl^ CD4-Cre and *Cdk6* ^fl/fl^ CD8+ T cells (**Figure 7B**). The analysis of previously published ChIP-seq experiments in lymphoid cells (12) revealed the presence of CDK6 at the promoter regions of *Socs1* and *Socs3* (**Figures 7C, D**). This implies that CDK6 regulates the expression of *Socs* genes as a direct chromatin-bound transcriptional regulator. Furthermore, CDK6 was also found to bind to promoter regions of *Mx2* and *Tap1*, but not *Mx1* (**Figures 7E, F**, **Figure S3I**). Based on these data we propose the following model: CDK6 promotes early IFN responses as well as their negative feedback loops. In the absence of CDK6, attenuated negative feedback regulation might compensate for the reduced early IFN response, which could contribute to unaffected anti-viral or anti-tumor immunity in the absence of T cell-intrinsic CDK6.

## Discussion

We here demonstrate that CDK6 in CD8+ T cells contributes to *in vitro* expansion and reduces mitochondrial respiration in CD8+ T cells. These changes do not translate into major alterations of CD8+ T cell effector functions *in vitro* and T cell-intrinsic loss of CDK6 does not impact on anti-viral and anti-tumor responses *in vivo.* This discrepancy might partially be due to the complex interference of CDK6 in the fine-tuning of IFN responses.

T cell-intrinsic CDK6 is required for *in vitro* proliferation of CD8+ T cells upon αCD3/αCD28 stimulation. This finding is in accordance with data showing that T cells depend on CDK6 for proliferation under different mitogenic conditions (5). *In vitro* expansion requires the kinase activity of CDK6 as the reduced proliferation extends to *Cdk6* ^K43M^ CD8+ T cells. CD8+ T cells produce IL-2, which stimulates their proliferation. Of note cultured *Cdk6* ^fl/fl^ CD4-Cre CD8+ T cells produce more IL-2 compared to *Cdk6* ^fl/fl^ controls, which excludes IL-2 as a source of the proliferation defect upon CDK6 deficiency. The impaired proliferation *in vitro* contrasts the *in vivo* situation, where we did not find effects of CDK6 deficiency on expansion of virus-specific CD8+ T cells upon LCMV infection. Similarly, we failed to observe significant differences in the absolute number of MC38 tumor-infiltrating CD8+ T cells. This led us to speculate that in contrast to the *in vitro* situation, T cell-intrinsic CDK6 expression is not required for expansion or infiltration of CD8+ T cells during anti-viral and anti-tumor responses *in vivo*. As CDK6 expression is regulated by STAT5 and significantly increases upon IL-2 stimulation (67), it is attractive to speculate that IL-2 cultured CD8+ T cells might react differently and become more dependent on CDK6 expression for proliferation.

Similar to CD8+ T cells, in steady-state the numbers of CD4+ T cells including Tregs were unchanged in *Cdk6* ^fl/fl^ CD4-Cre mice. This contrasts previous reports showing that Tregs are particularly sensitive to CDK4/6 inhibitor treatment (24, 25, 68). CDK4/6 inhibitors target CDK4 and CDK6 kinases while we concentrate on the sole loss of CDK6, which may explain this apparent contradiction.

CDK6 suppresses metabolic pathways in a kinase-dependent manner, including the TCA cycle. In accordance with the findings of our RNA-seq analysis, Seahorse experiments confirmed the repressive effect of CDK6 on mitochondrial respiration *in vitro*. Pancreatic cancer cells react to CDK4/6 inhibition with enhanced oxidative phosphorylation, associated with an RB-dependent increase in mitochondrial mass and mTORC1 activation (41). Our data support the concept of CDK6 being involved in the regulation of mitochondrial metabolism and extends it to non-transformed cells. The detailed underlying molecular mechanisms however remain to be determined. Other metabolic effects of CDK6 were described for T-ALL cells, where CDK6 inhibits glycolysis in a kinase-dependent manner (38). We did not observe an effect of T cell-intrinsic loss of CDK6 on the glycolytic activity of CD8+ T cells. Therefore, the role of CDK6 in glycolysis might be specific for transformed cells and was indicated to be dependent on high levels of cylcinD3-CDK6 complexes (38).

Mitochondrial ATP production is required for early effector T cell activation and proliferation (46, 49). The increased mitochondrial respiration did not compensate for the *in vitro* expansion defect in *Cdk6* ^fl/fl^ CD4-Cre CD8+ T cells. We also observed a trend for increased mitochondrial SRC in CDK6-deficient CD8+ T cells. The significance of SRC and mitochondrial biogenesis for CD8+ T cell effector function is discussed controversially (48, 69). We failed to observe a link between mitochondrial metabolic activity and CD8+ T cell-mediated cytotoxicity and production of the effector cytokines IFN-γ and TNF-α *in vitro*. While T cell-intrinsic CDK6 suppresses mitochondrial respiration in CD8+ T cells, this is not associated with a major difference in activation or effector function.

IL-2 production *in vitro* was increased in CDK6-deficient CD4+ and CD8+ T cells. It is currently enigmatic whether the differences in IL-2 production relate to differences in metabolic activity of CD8+ T cells. CDK4/6 inhibitor treatment enhances IL-2 production upon αCD3/αCD28 stimulation by the CD4+ Jurkat T cell line and human CD4+ T cells. This phenomenon could be recapitulated by CDK6 knockdown experiments in Jurkat cells and was linked to de-repression of NFAT signaling (25). We extend this finding showing that CDK6 plays a role in the suppression of IL-2 production in both CD4+ and CD8+ T cells.

CDK4/6 inhibitor treatment promotes T cell-mediated anti-tumor immunity by different means (24, 25, 27). We here show that T cell-intrinsic loss of CDK6 does not suffice to cause enhanced anti-tumor immunity against MC38 tumors *in vivo*. This suggests that the effects of CDK4/6 inhibitors predominantly relate to T cell-extrinsic mechanisms, including increased antigen-presentation capacity of tumor cells or APCs that boost anti-tumor immunity (24, 25, 27). The potential need for combined inhibition of CDK4 and CDK6 kinase activities represents another possibility that might explain why T cell-intrinsic CDK6-deficiency does not have an impact on anti-tumor immunity.

Of note the *Cdk6* ^fl/fl^ CD4-Cre mouse model not exclusively deletes CDK6 in CD8+ T cells. We can therefore not unequivocally rule out that a complex pattern of phenotypes occurring in different T cell subsets accounts for the lack of any effects *in vivo*. To clearly disentangle any compensatory effect of CDK6-deficiency on different T cell populations, analysis of different Cre – lines that specifically delete in distinct T cell subsets is required. MC38 tumors from *Cdk6* ^fl/fl^ CD4-Cre mice harbor lower numbers of infiltrating total CD4+ T cells as well as Tregs. In contrast to previous studies using the CDK4/6 inhibitor Abemaciclib in a breast cancer model as well as in CT-26 tumor-bearing mice (24), we did not observe a selective decrease in the frequency of Tregs within CD4+ T cells. This may again be related to the fact that Abemaciclib not only targets CDK6 but also CDK4. Most importantly, the reduced tumor-infiltrating CD4+ T cell numbers do not result in an altered MC38 tumor growth. It is attractive to speculate that a reduction in pro-tumorigenic/immunosuppressive Tregs is counterbalanced by the reduction of other anti-tumorigenic CD4+ T cell subsets that support CD8+ T cell anti-tumor activity.

CDK6 is involved in interferon signaling as evident from the RNA-seq analysis. ISG expression is impaired in the absence of CDK6, indicating a contribution of CDK6 for tonic IFN signaling under steady-state conditions in CD8+ T cells. CDK4/6 inhibitor treatment enhances MHC class I levels on tumors cells, which was linked to increased type III IFN production following DNMT1 downregulation and hypomethylation of endogenous retroviral elements (24). CDK6 transcriptionally regulates DNMT3B in BCR-ABL transformed cells (70). However, the regulation of DNMTs by CDK6 appears to be cell type-specific or dependent on transformation, as we did not observe a significant deregulation of DNMTs in CDK6-deficient steady-state CD8+ T cells (data not shown). Consequently, the re-expression of retroviral elements does not necessarily extend to CDK6-deficient CD8+ T cells, where we found the “Class I MHC mediated antigen processing and presentation” pathway to be downregulated (**Figure 2D**). Apart from tonic IFN signaling, we also observed partially reduced expression of ISGs upon short-term IFN-β stimulation of CDK6-deficient CD8+ T cells. In summary, these data indicate that CDK6 contributes to early type I IFN responses.

CDK6 cooperates with the Janus kinase/signal transducers and activators of transcription (JAK/STAT) signaling pathway (8, 12). As such the direct interaction of CDK6 and STAT3 drives expression of the cell cycle inhibitor and tumor suppressor p16^INK4a^ (8). CDK6 ChIP-seq experiments in transformed pro B cell lines uncovered around 30.000 specific binding sites for CDK6 on chromatin (12). Of note, the CDK6 chromatin binding profiles in these lymphoid cells extend to other cell types and generally mark CDK6-regulated genes. CDK6 ChIP-seq peaks are found at the promoter sites of genes, including Aurora kinases and Akt1-3, which lack transcriptional regulation by CDK6 in transformed B cells, but are subject to CDK6-dependent regulation in transformed human and murine myeloid cells (11, 12). Dependent on the cellular context and stimulation condition chromatin-bound CDK6 may modulate gene expression. Our ChIP-seq data (12) also show that CDK6 is present at the promoter regions of *Mx2* and *Tap1*. CDK6 lacks a DNA binding domain and depends on the interaction with transcription factors for regulation of gene expression (8, 9, 12, 15). As ISG expression was partially attenuated in CDK6-deficient CD8+ T cells upon type I IFN stimulation, it is attractive to speculate that CDK6 cooperates with transcription factors downstream of type I IFN signaling to directly drive transcription of some ISGs. In addition to interfering with JAK/STAT signaling, CDK6 directly interacts with NF-κB signaling. CDK6 acts as a nuclear co-factor and downregulates NF-κB inhibitors thereby contributing to the production of different inflammatory cytokines (13, 15). NF-κB has a complex role in the regulation of ISGs, suppressing some ISGs, including *Mx1, Mx2* and *Tap1*, while enhancing others (71). The complex and diverse interplay of CDK6 with multiple signaling pathways might contribute to a regulation of IFN responses at multiple levels.

Type I IFNs play an important role during anti-viral responses and T cell-intrinsic IFN signaling regulates CD8+ T cell responses in LCMV infection (43, 54–59). In line, *Ifnar1* ^fl/fl^ CD4-Cre CD8+ T cells show an impaired activation and antigen-specific response in chronic LCMV Clone 13 infections (43). Despite the impaired type I IFN response observed in CDK6-deficient CD8+ T cells *in vitro*, *in vivo* we failed to observe differences in LCMV Clone 13-induced CD8+ T cell responses between *Cdk6* ^fl/fl^ CD4-Cre and *Cdk6* ^fl/fl^ mice.

Interferon responses are inducing a negative feedback loop that involves SOCS proteins (62, 63). SOCS1 and SOCS3 are induced by type I IFNs and inhibit JAK/STAT signaling by binding the IFN receptor or associated JAKs (62, 64, 65, 72–75). The analysis of previously performed CDK6 ChIP-seq experiments (12) uncovered that CDK6 binds to *Socs1* and *Socs3* promoter regions, which indicates a direct transcriptional regulation and may explain why *Socs1* and *Socs3* are downregulated in CDK6-deficient CD8+ T cells. This proposes a model where CDK6 is involved in the regulation of negative feedback loops in the JAK/STAT signaling pathway to fine-tune cytokine responses. Overall, it is attractive to speculate that CDK6 has a dual role in regulating IFN signaling in CD8+ T cells. It promotes the tonic and/or early stimulated type I IFN response, but at the same time stimulates a negative feedback loop by up-regulation of SOCS proteins. In the absence of CDK6, the attenuated initial ISG response is counteracted by a weakened negative feedback loop, which might contribute to the lack of obvious *in vivo* phenotypes upon viral or tumor challenge.

## Data Availability Statement

The datasets presented in this study can be found in online repositories. The names of the repository/repositories and accession number(s) can be found below: https://www.ncbi.nlm.nih.gov/geo/, GSE164002.

## Ethics Statement

Animal experiments were approved by the Ethics and Animal Welfare Committee of the University of Veterinary Medicine Vienna and the national authority (Austrian Federal Ministry of Science and Research) according to §§ 26ff. of Animal Experiments Act, Tierversuchsgesetz 2012—TVG 2012, under licenses BMBWF-68.205/0174-V/3b/2018 (GZ 2020-0.050.677) and BMWFW-66.009/0361-WF/V/3b/2017 (GZ 2021-0.009.992).

## Author Contributions

KK, AW-S, BA, FL, and VK performed and analyzed the experiments. RG performed the bioinformatic analysis. DG provided scientific support. VS supervised the study. VS and AB provided resources. KK, AW-S and VS wrote the manuscript. All authors contributed to the article and approved the submitted version.

## Funding

The work was supported by the Austrian Science Fund FWF funded Ph.D. program, “Inflammation and Immunity” FWF W1212, FWF grant SFB-F06107 and the European Research Council (ERC) under the European Union’s Horizon 2020 research and innovation program grant agreement 694354.

## Conflict of Interest

The authors declare that the research was conducted in the absence of any commercial or financial relationships that could be construed as a potential conflict of interest.

## Abbreviations

AICD, Activation-induced cell death; ALT, Alanine aminotransferase; AP-1, Activator protein 1; APC, Antigen-presenting cell; CDK6, Cyclin-dependent kinase 6; DNMT, DNA methyltransferase; ECAR, Extracellular acidification rates; FcγR, Fc-gamma receptor; IFN, Interferon; ISG, Interferon-stimulated gene; JAK, Janus kinase; LCMV, Lymphocytic choriomeningitis virus; NFAT, Nuclear factor of activated T-cells; NF-κB, Nuclear factor kappa B; ns, not significant; OCR, Oxygen consumption rates; pLN, peripheral lymph nodes; SOCS,Suppressor of cytokine signaling; SRC, Spare respiratory capacity; STAT, Signal transducer and activator of transcription; TNF-α, Tumor Necrosis Factor alpha; Tregs, regulatory T cells.
