# Supplementary material for: T Cell-Intrinsic CDK6 Is Dispensable for Anti-Viral and Anti-Tumor Responses In Vivo
Source: Front Immunol. 2021 Jun 24;12:650977. doi: 10.3389/fimmu.2021.650977 (PMC8264666; doi:10.3389/fimmu.2021.650977)
Supplement: Supplementary file 1 [file DataSheet_1.pdf]

Supplementary Material

T cell-intrinsic CDK6 is dispensable for anti-viral and anti-tumor responses *in vivo*

Klara Klein[1], Agnieszka Witalisz-Siepracka[1,2], Dagmar Gotthardt[1], Benedikt Agerer[3], Felix Locker[4], Reinhard Grausenburger[1], Vanessa Maria Knab[1], Andreas Bergthaler[3], Veronika Sexl[1*]

[1] Institute of Pharmacology and Toxicology, University of Veterinary Medicine Vienna, Vienna, Austria.

[2] Karl Landsteiner University of Health Sciences, Department of Pharmacology, Physiology and Microbiology, Division Pharmacology, Krems, Austria.

[3] CeMM Research Center for Molecular Medicine of the Austrian Academy of Sciences, Vienna, Austria.

[4] Institute of Physiology, Pathophysiology and Biophysics, University of Veterinary Medicine, Vienna, Austria.

*** Correspondence:** Email: veronika.sexl@vetmeduni.ac.at

Supplementary Figures 1-6

Figure S1. Loss of CDK6 and its kinase activity deregulates major cellular processes as well as expression of interferon-stimulated genes in steady-state CD8+ T cells. Splenic CD3+CD8+ T cells from WT, *Cdk6*$^{-/-}$ and *Cdk6*^{K43M} mice were sorted *ex vivo* and lysed for RNA-sequencing. Three biological replicates (each pooled from two mice) were prepared per genotype. (A-B) Venn diagrams of significantly (FDR < 0.05, >1.25x fold change) upregulated (A) or downregulated (B) genes in *Cdk6*$^{-/-}$ and *Cdk6*^{K43M} vs. WT CD8+ T cells are shown. (C-D) Pathway analysis was performed using the Reactome Pathway tool with genes that were significantly upregulated (C) or downregulated (D) in *Cdk6*^{K43M} compared to WT CD8+ T cells. Significant pathways (FDR<0.05) are ranked by fold enrichment. Bar graphs show the top 20 non-redundant Reactome pathways in (C), while all significant non-redundant pathways are depicted in (D). (E) A Venn diagram overlapping significantly (FDR < 0.05, >1.25x fold change) downregulated genes in *Cdk6*$^{-/-}$ vs. WT CD8+ T cells with core type I and II ISGs (according to interferome database) is shown. Genes contained in the intersections are listed.

Genes that were part of the Reactome pathway "interferon signaling" (Fig. 2D) are indicated in bold, genes that were also downregulated in *Cdk6*^{K43M} vs. WT CD8+ T cells are indicated in blue.

Figure S2. Loss of CDK6 impairs CD8+ T cell proliferation *in vitro*. (A) The frequencies of CD4/CD8 double-negative (DN), CD4/CD8 double-positive (DP), CD4 single-positive (CD4 SP) and CD8 single-positive (CD8 SP) thymocytes were analyzed by flow cytometry in *Cdk6*$^{fl/fl}$ CD4-Cre mice and *Cdk6*$^{fl/fl}$ controls (n=4-7). (B-C) Naïve CD8+ T cells were isolated from spleens of *Cdk6*$^{fl/fl}$ and *Cdk6*$^{fl/fl}$ CD4-Cre mice and labelled with CFSE. CFSE proliferation dye dilution was analyzed after 72h of culture by flow cytometry. (B) Representative histograms and (C) quantifications of the percentages of cells in each of the CFSE peaks/generations (numbered from 1-7, where 1 is the population with the lowest proliferation and 7 is the population with the highest amount of cell divisions) are shown (n=3). (D-E) Splenic naïve CD8+ T cells were isolated from *Cdk6*$^{fl/fl}$ and *Cdk6*$^{fl/fl}$ CD4-Cre mice and activated with αCD3/αCD28 for 48h in the presence of IL-2. Expression of CD69, CD25, CD44, CD62L and Ki67 were analyzed by flow cytometry. (D) Representative histograms for each marker on cultured *Cdk6*$^{fl/fl}$ (in blue) and *Cdk6*$^{fl/fl}$ CD4-Cre CD8+ T cells (in red) and a negative staining control (FMO = fluorescence minus one) (in black) are shown. (E) Quantifications of expression levels as relative median fluorescence intensity (MFI) are shown, calculated as fold of change over WT levels (n=3-8). (A,C,E) Bar graphs represent mean ± SEM, from one experiment (C) or pooled from 2-3 independent experiments. ***p<0.001,**p<0.01, *p<0.05, unpaired t-test.

Figure S3. CDK6-deficient CD8+ T cells show increased IL-2 production *in vitro*. (A) Splenic naïve CD8+ T cells were isolated from *Cdk6*$^{fl/fl}$ and *Cdk6*$^{fl/fl}$ CD4-Cre mice, activated with αCD3/αCD28 for 48h and cultured in the presence of IL-2 for a total of six days. Percentages of naïve (CD62L+CD44-), central memory (CD62L+CD44+) and effector memory (CD62L-CD44+) CD8+ T cells were analyzed after six days of culture by flow cytometry (n=4). (B) Percentages of CD62L+CD44-, CD62L+CD44+, CD62L-CD44+ splenic CD8+ T cells were analyzed *ex vivo* by flow cytometry in *Cdk6*$^{fl/fl}$ and *Cdk6*$^{fl/fl}$ CD4-Cre mice (n=7-9). (C) Percentages of CD69+ CD8+ T cells (left panel) and CD69 expression levels (median fluorescence intensity (MFI)) on CD69+ CD8+ T cells (right panel) were analyzed *ex vivo* by flow cytometry in spleens of *Cdk6*$^{fl/fl}$ and *Cdk6*$^{fl/fl}$ CD4-Cre mice (n=6). (D) Percentages of Ki67+ CD8+ T cells (left panel) and Ki67 levels (MFI) on Ki67+ CD8+ T cells (right panel) were analyzed *ex vivo* by flow cytometry in spleens of *Cdk6*$^{fl/fl}$ and *Cdk6*$^{fl/fl}$ CD4-Cre mice (n=6). (E-G) Isolated naïve CD8+ T cells were cultured as described in (A). Cultured

Cdk6$^{fl/fl}$ and *Cdk6*$^{fl/fl}$ CD4-Cre CD8+ T cells were kept unstimulated or stimulated with PMA/Iono(mycin) for 4h and percentages of IFN-γ (E), TNF-α (F) and IL-2 (G) positive CD8+ T cells were analyzed by intracellular flow cytometric staining (n=4-5). (H) *Cdk6*$^{fl/fl}$ and *Cdk6*$^{fl/fl}$ CD4-Cre mice were subcutaneously injected into both flanks with 10^6 MC38 tumor cells. The weights of the isolated tumors were determined 16 days post injection (n=4-5 mice per genotype (8-10 tumors per genotype)). (I) CDK6 ChIP-seq was previously performed in p185$^{BCR\text{-}ABL}$-transformed B cell lines (GSE113752). CDK6 ChIP-seq results from three biological replicates (WT1-3) at the *Mx1* locus are shown. Corresponding input samples are shown as controls. (A-G) Bar graphs represent mean ± SEM, pooled from 2-3 independent experiments. (H) Bar graph represents mean ± SEM with symbols representing values from individual tumors from one experiment. ***p<0.001, **p<0.01, *p<0.05, unpaired t-test.

Figure S4. T cell-intrinsic loss of CDK6 is associated with a higher percentage of naïve and lower percentage of effector memory CD4+ T cells in steady-state. (A-B) Total numbers (left panel) and percentages (right panel) of CD4+ T cells in spleens (n=7-9) (A) and peripheral lymph nodes (pLNs) (n=6) (B) were analyzed *ex vivo* by flow cytometry in *Cdk6*$^{fl/fl}$ and *Cdk6*$^{fl/fl}$ CD4-Cre mice. (C-D) Total numbers (left panel) and percentages (right panel) of regulatory T cells (Tregs) (CD25+Foxp3+CD4+ T cells) in spleens (n=6) (C) and pLNs (n=6) (D) were analyzed *ex vivo* by flow cytometry in *Cdk6*$^{fl/fl}$ and *Cdk6*$^{fl/fl}$ CD4-Cre mice. (E-F) Percentages of CD62L+CD44-, CD62L+CD44+, CD62L-CD44+ CD4+ T cells were analyzed in spleens (n=7-9) (E) and pLNs (n=6) (F) *ex vivo* by flow cytometry in *Cdk6*$^{fl/fl}$ and *Cdk6*$^{fl/fl}$ CD4-Cre mice. Total cell numbers in pLNs refer to total cell numbers in six pLNs (2x inguinal, 4x axillary LNs). (A-B,E-F) Bar graphs represent mean ± SEM, pooled from 2 independent experiments. (C-D) Bar graphs represent mean ± SEM from one experiment. **p<0.01, *p<0.05, unpaired t-test.

Figure S5. Loss of CDK6 is associated with increased IL-2 production by activated CD4+ T cells. (A-F) Naïve CD4+ T cells were sorted from spleens and pLNs of *Cdk6*$^{fl/fl}$ and *Cdk6*$^{fl/fl}$ CD4-Cre mice and activated with αCD3/αCD28 for 72h in the presence of IL-2. (A-B) Cultured *Cdk6*$^{fl/fl}$ and *Cdk6*$^{fl/fl}$ CD4-Cre CD4+ T cells were kept unstimulated or stimulated with PMA/Iono(mycin) for 4h and percentages of IL-2 positive CD4+ T cells were analyzed by intracellular flow cytometric staining (n=3). Representative dot plots (A) and the quantification (B) are shown. (C-F) Cell surface expression of the activation markers CD25 and CD69 was analyzed on cultured CD4+ T cells. Representative histograms for CD25 (C) and CD69 (E) expression on cultured *Cdk6*$^{fl/fl}$ (in blue) and *Cdk6*$^{fl/fl}$ CD4-Cre

CD4+ T cells (in red) and a negative staining control (FMO) (in black) as well as quantifications of CD25 (D) and CD69 (F) expression levels are shown (n=3). (B,D,F) Bar graphs represent mean ± SEM from one experiment. ***p<0.001, **p<0.01, unpaired t-test.

Figure S6. T cell-intrinsic loss of CDK6 is associated with decreased numbers of MC38 tumor-infiltrating CD4+ T cells. (A-F) 10^6 MC38 tumor cells were subcutaneously injected into both flanks of *Cdk6*$^{fl/fl}$ and *Cdk6*$^{fl/fl}$ CD4-Cre mice. Frozen tumor cell suspensions were prepared and analyzed by flow cytometry. (A) Absolute numbers (normalized to tumor weight) (left panel) and percentages of tumor-infiltrating CD4+ T cells (within CD45+ cells) (right panel) were analyzed by flow cytometry (n=4-5). (B) Absolute numbers (normalized to tumor weight) (left panel) and percentages of tumor-infiltrating Tregs (CD25+Foxp3+CD4+ T cells) (within CD4+ T cells) (right panel) were analyzed by flow cytometry (n=4-5). (C) Percentages of CD69+ CD4+ T cells (left panel) and CD69 expression levels (MFI) on tumor-infiltrating CD4+ T cells (right panel) were analyzed by flow cytometry (n=4-5). (D) Percentages of CD69+ Tregs (left panel) and CD69 expression levels (MFI) on Tregs (right panel) were analyzed by flow cytometry (n=4-5). (E-F) Percentages of CD62L+CD44-, CD62L+CD44+, CD62L-CD44+ CD4+ T cells (E) and Tregs (F) were analyzed by flow cytometry (n=4-5). (A-D) Bar graphs represent mean ± SEM with symbols representing values from individual tumors from one experiment. (E-F) Bar graphs represent mean ± SEM from one experiment. *p<0.05, unpaired t-test.

Figure S1.

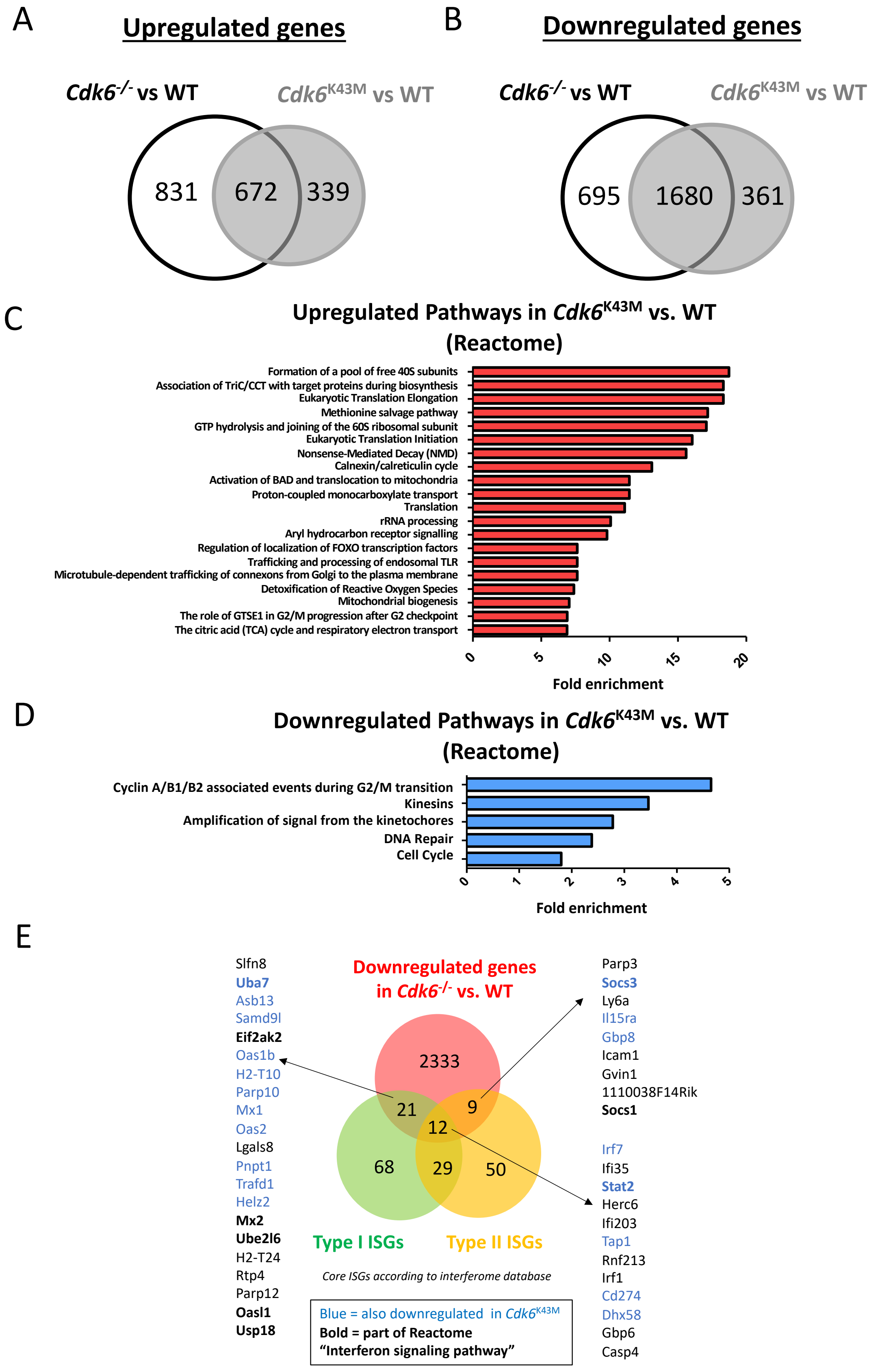
A
Upregulated genes
Cdk6-/- vs WT
Cdk6K43M vs WT
831
672
339
B
Downregulated genes
Cdk6-/- vs WT
Cdk6K43M vs WT
695
1680
361
C
Upregulated Pathways in Cdk6K43M vs. WT (Reactome)
Formation of a pool of free 40S subunits
Association of TriC/CCT with target proteins during biosynthesis
Eukaryotic Translation Elongation
Methionine salvage pathway
GTP hydrolysis and joining of the 60S ribosomal subunit
Eukaryotic Translation Initiation
Nonsense-Mediated Decay (NMD)
Calnexin/calreticulin cycle
Activation of BAD and translocation to mitochondria
Proton-coupled monocarboxylate transport
Translation
rRNA processing
Aryl hydrocarbon receptor signalling
Regulation of localization of FOXO transcription factors
Trafficking and processing of endosomal TLR
Microtubule-dependent trafficking of connexons from Golgi to the plasma membrane
Detoxification of Reactive Oxygen Species
Mitochondrial biogenesis
The role of GTSE1 in G2/M progression after G2 checkpoint
The citric acid (TCA) cycle and respiratory electron transport
0
5
10
15
20
Fold enrichment
D
Downregulated Pathways in Cdk6K43M vs. WT (Reactome)
Cyclin A/B1/B2 associated events during G2/M transition
Kinesins
Amplification of signal from the kinetochores
DNA Repair
Cell Cycle
0
1
2
3
4
5
Fold enrichment
E
Slfn8
Uba7
Asb13
Samd9l
Eif2ak2
Oas1b
H2-T10
Parp10
Mx1
Oas2
Lgals8
Pnpt1
Trafd1
Helz2
Mx2
Ube2l6
H2-T24
Rtp4
Parp12
Oasl1
Usp18
Downregulated genes in Cdk6-/- vs. WT
2333
21
9
12
68
29
50
Type I ISGs
Type II ISGs
Core ISGs according to interferome database
Blue = also downregulated in Cdk6K43M
Bold = part of Reactome "Interferon signaling pathway"
Parp3
Socs3
Ly6a
Il15ra
Gbp8
Icam1
Gvin1
1110038F14Rik
Socs1
Irf7
Ifi35
Stat2
Herc6
Ifi203
Tap1
Rnf213
Irf1
Cd274
Dhx58
Gbp6
Casp4

Figure S2.

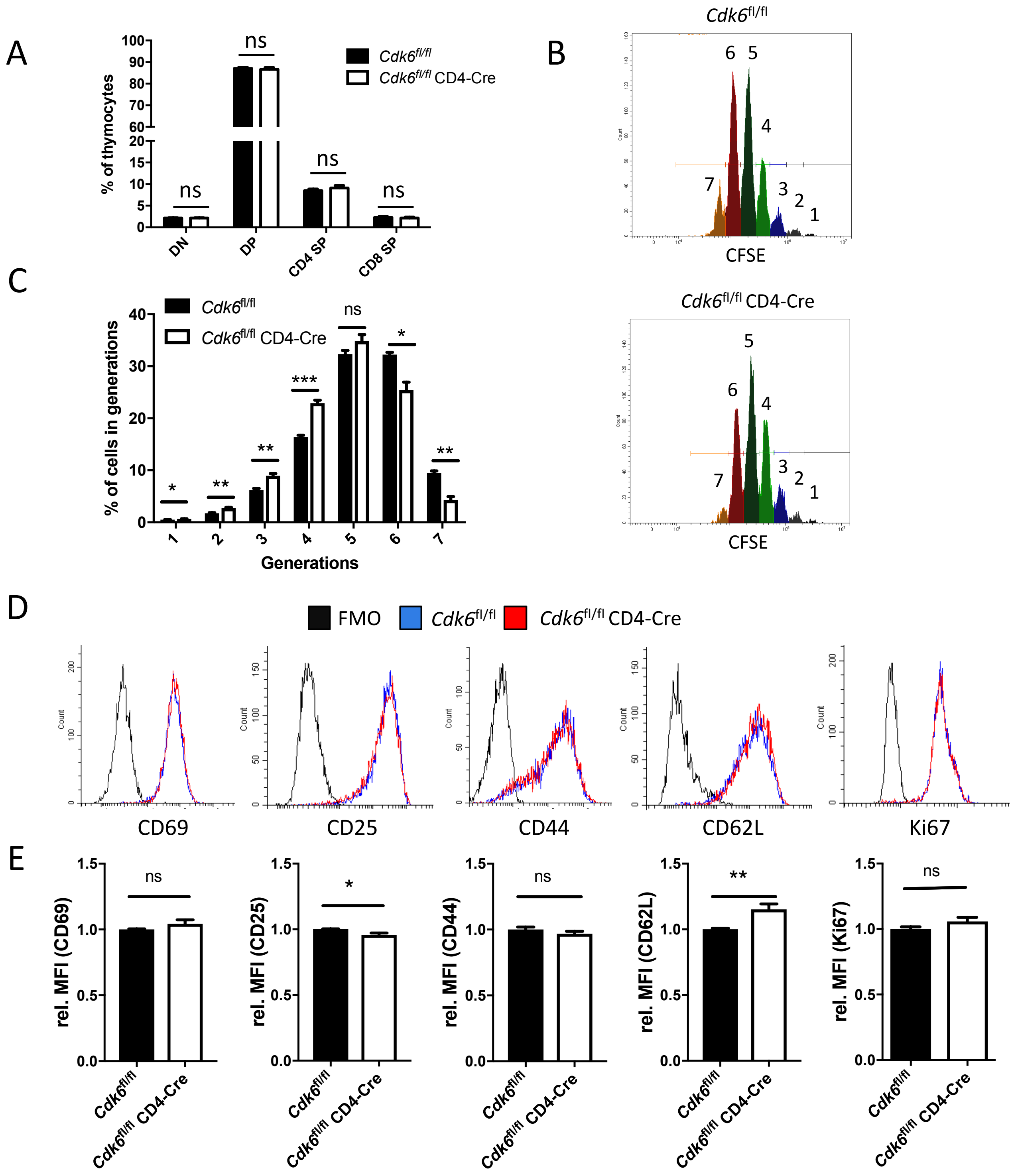
A
% of thymocytes
ns
DN
DP
CD4 SP
CD8 SP
Cdk6fl/fl
Cdk6fl/fl CD4-Cre
B
Cdk6fl/fl
CFSE
Cdk6fl/fl CD4-Cre
CFSE
C
% of cells in generations
Generations
ns
*
**
***
D
FMO
Cdk6fl/fl
Cdk6fl/fl CD4-Cre
CD69
CD25
CD44
CD62L
Ki67
E
rel. MFI (CD69)
rel. MFI (CD25)
rel. MFI (CD44)
rel. MFI (CD62L)
rel. MFI (Ki67)

Figure S3.

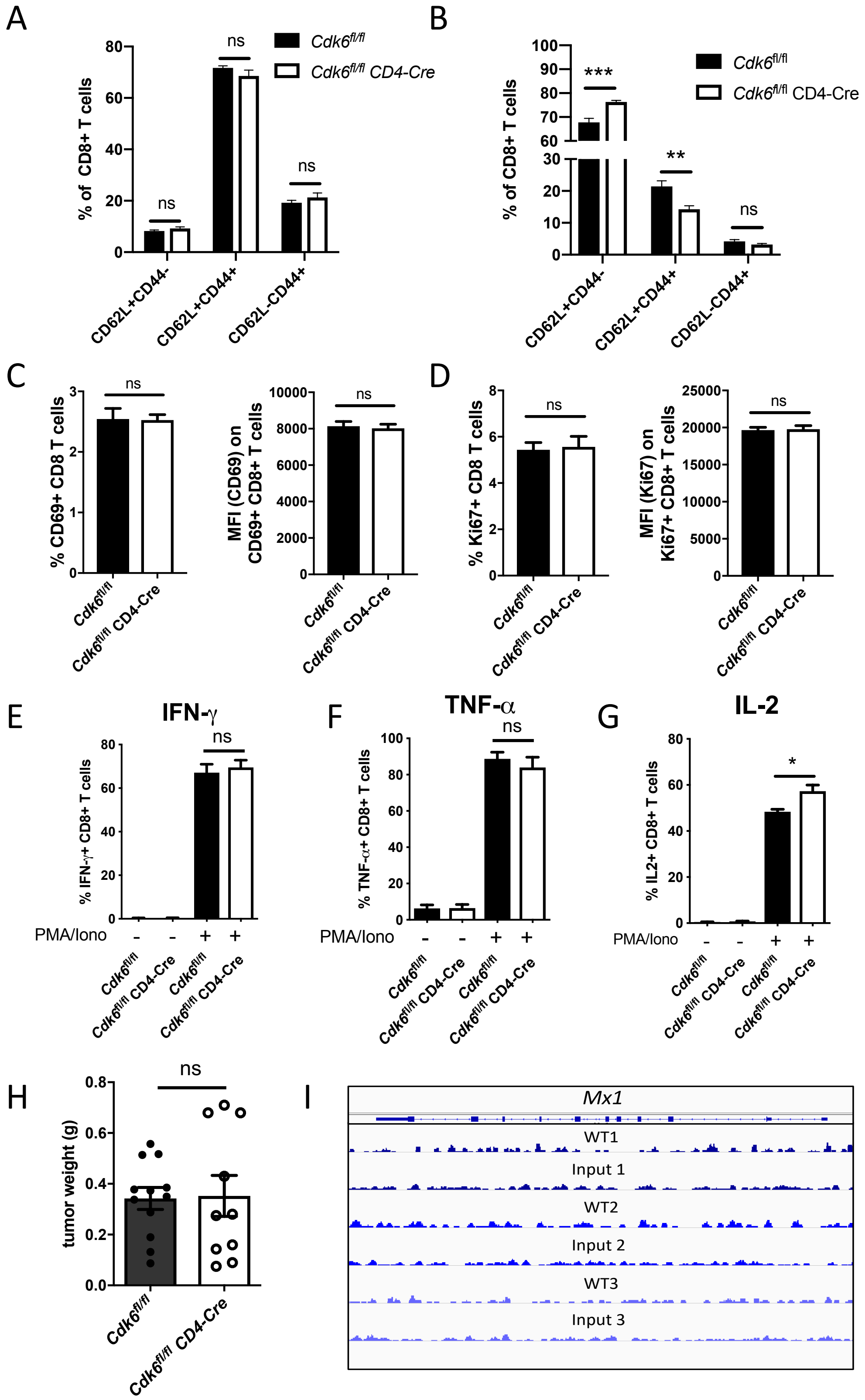
A
% of CD8+ T cells
Cdk6fl/fl
Cdk6fl/fl CD4-Cre
ns
CD62L+CD44-
CD62L+CD44+
CD62L-CD44+
B
% of CD8+ T cells
***
**
ns
C
% CD69+ CD8 T cells
MFI (CD69) on CD69+ CD8+ T cells
D
% Ki67+ CD8 T cells
MFI (Ki67) on Ki67+ CD8+ T cells
E
IFN-γ
% IFN-γ+ CD8+ T cells
PMA/Iono
F
TNF-α
% TNF-α+ CD8+ T cells
G
IL-2
% IL2+ CD8+ T cells
*
H
tumor weight (g)
I
Mx1
WT1
Input 1
WT2
Input 2
WT3
Input 3

Figure S4.

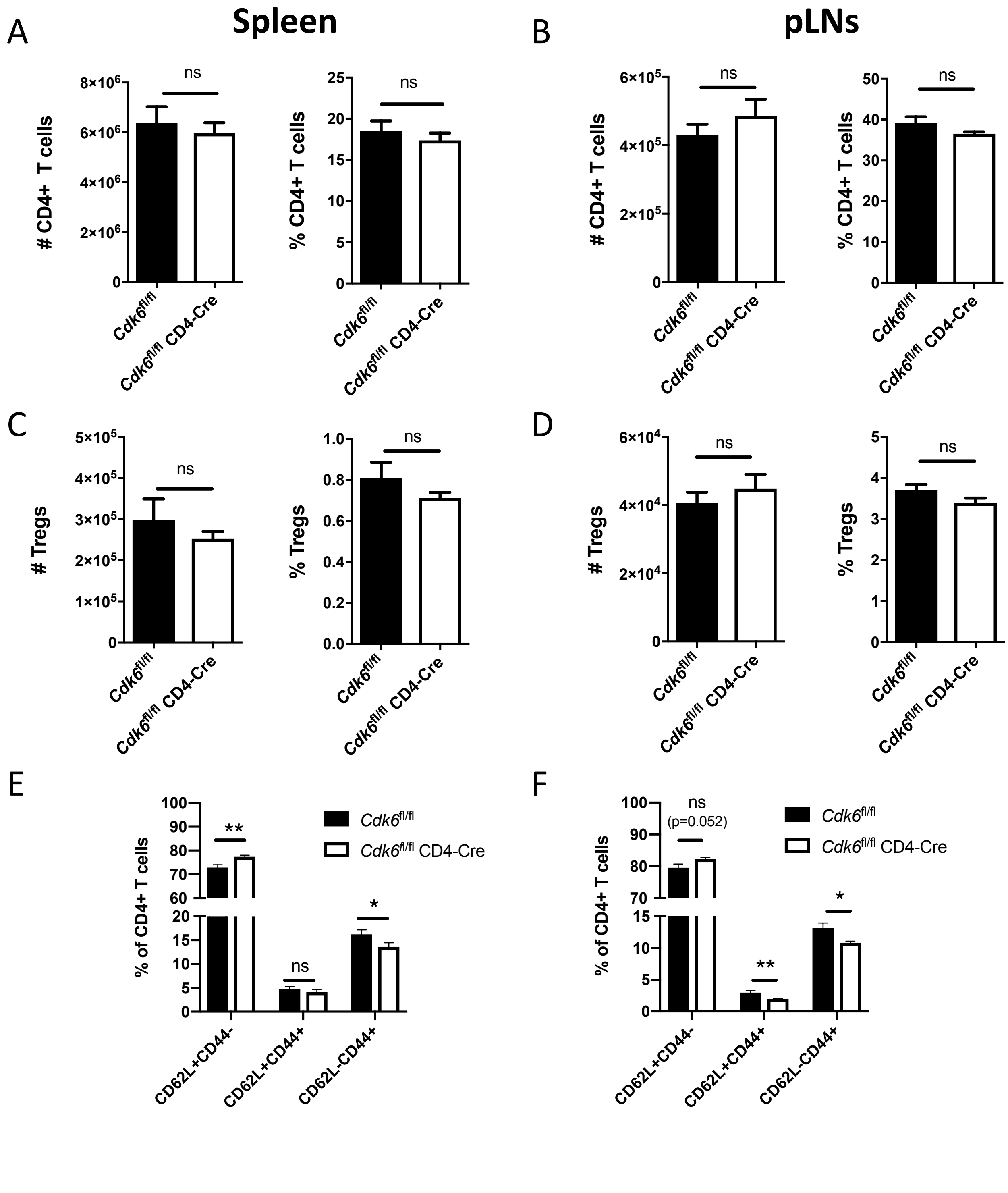
A
Spleen
B
pLNs
ns
CD4+ T cells
% CD4+ T cells
# CD4+ T cells
% CD4+ T cells
Cdk6fl/fl
Cdk6fl/fl CD4-Cre
C
D
Tregs
% Tregs
E
F
% of CD4+ T cells
**
*
ns (p=0.052)
CD62L+CD44-
CD62L+CD44+
CD62L-CD44+

Figure S5.

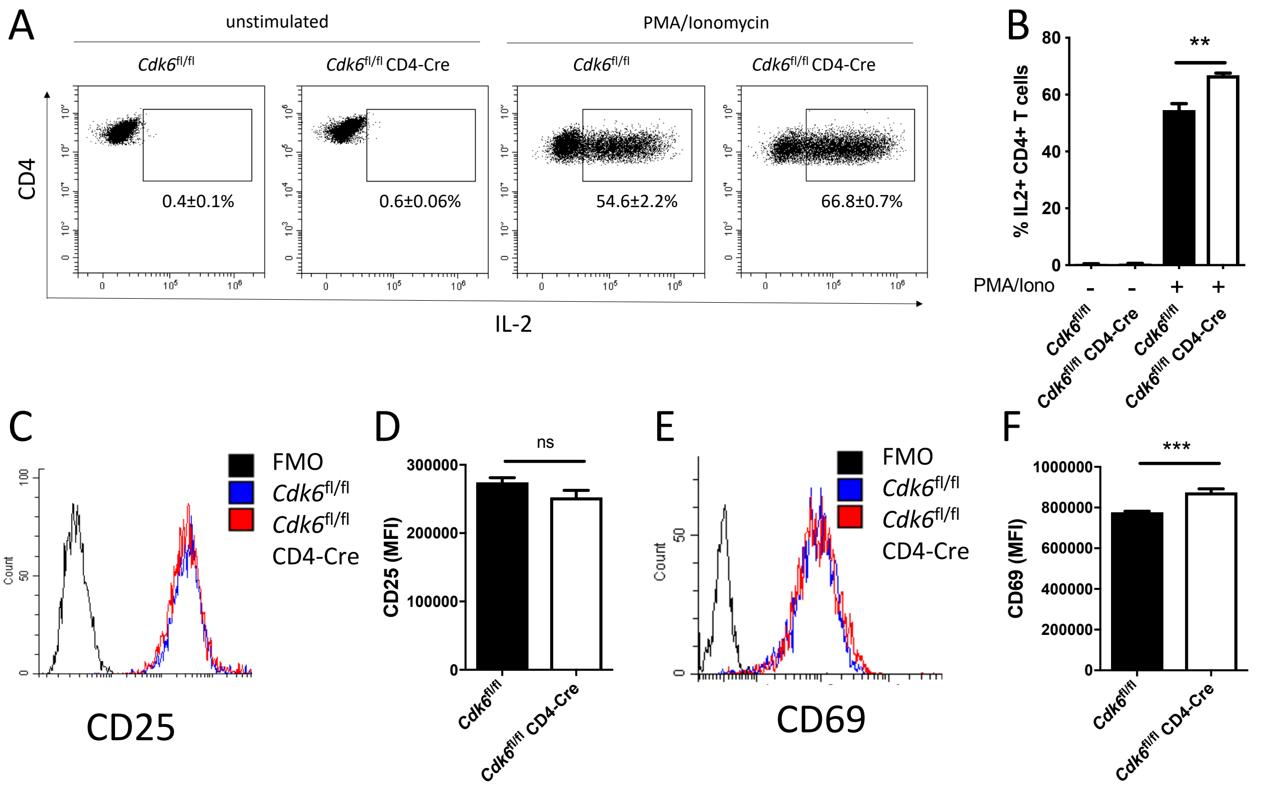

A
unstimulated
PMA/Ionomycin
Cdk6fl/fl
Cdk6fl/fl CD4-Cre
CD4
IL-2
0.4±0.1%
0.6±0.06%
54.6±2.2%
66.8±0.7%
B
% IL2+ CD4+ T cells
**
PMA/Iono
Cdk6fl/fl
Cdk6fl/fl CD4-Cre
C
FMO
Cdk6fl/fl
Cdk6fl/fl CD4-Cre
Count
CD25
D
ns
CD25 (MFI)
E
CD69
F
***
CD69 (MFI)

Figure S6.

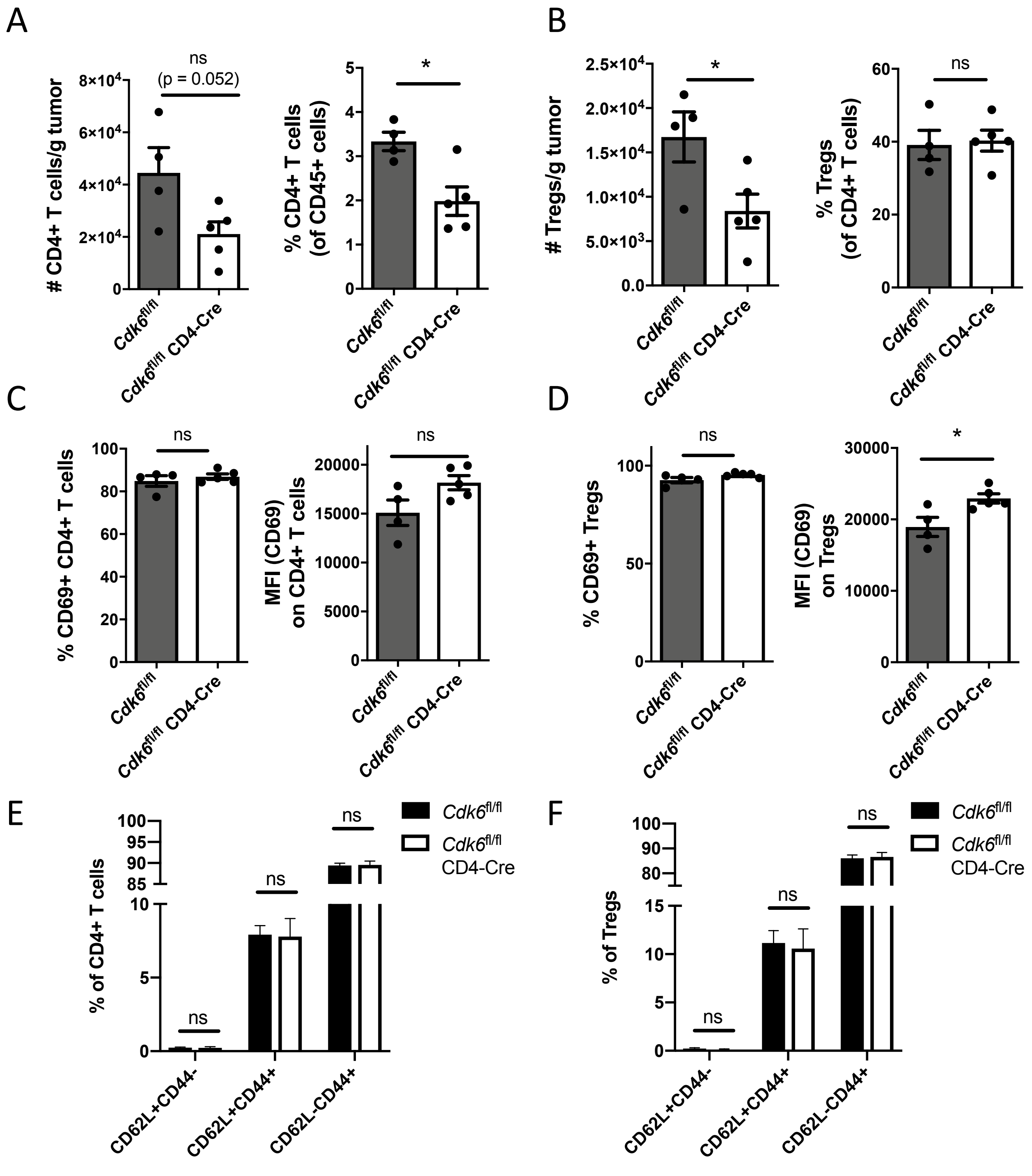
A
B
C
D
E
F
ns
(p = 0.052)
*
ns
CD4+ T cells/g tumor
% CD4+ T cells (of CD45+ cells)
# Tregs/g tumor
% Tregs (of CD4+ T cells)
% CD69+ CD4+ T cells
MFI (CD69) on CD4+ T cells
% CD69+ Tregs
MFI (CD69) on Tregs
% of CD4+ T cells
% of Tregs
Cdk6fl/fl
Cdk6fl/fl CD4-Cre
CD62L+CD44-
CD62L+CD44+
CD62L-CD44+
